# Lipid/water interface of galactolipid bilayers in different lyotropic liquid-crystalline phases

**DOI:** 10.3389/fmolb.2022.958537

**Published:** 2022-08-15

**Authors:** Jakub Hryc, Robert Szczelina, Michal Markiewicz, Marta Pasenkiewicz-Gierula

**Affiliations:** ^1^ Faculty of Biochemistry, Biophysics and Biotechnology, Jagiellonian University, Krakow, Poland; ^2^ Faculty of Mathematics and Computer Science, Jagiellonian University, Krakow, Poland

**Keywords:** monogalactolipid, digalactolipid, bilayer, stalk structure, inverse hexagonal phase, inter-lipid interactions, interaction network

## Abstract

In this study, carried out using computational methods, the organisation of the lipid/water interface of bilayers composed of galactolipids with both α-linolenoyl acyl chains is analysed and compared in three different lyotropic liquid-crystalline phases. These systems include the monogalactosyldiglyceride (MGDG) and digalactosyldiglyceride (DGDG) bilayers in the lamellar phase, the MGDG double bilayer during stalk phase formation and the inverse hexagonal MGDG phase. For each system, lipid-water and direct and water-mediated lipid-lipid interactions between the lipids of one bilayer leaflet and those of two apposing leaflets at the onset of new phase (stalk) formation, are identified. A network of interactions between DGDG molecules and its topological properties are derived and compared to those for the MGDG bilayer.

## 1 Introduction

Biological membranes (biomembranes) surround each cell and cell organelle. Their fundamental structural element is a lipid matrix that also plays the role of a selective permeability barrier. The biological functions that biomembranes can fulfil depend on the lipid composition of the matrix. The composition can vary within a wide range and determines the types and strength of intermolecular interactions and the molecular dynamics of lipids. Subsequently, it determines the physicochemical, biophysical, mechanical and other properties of the matrix and thus of the biomembrane. As the lipid matrix is an intricate system, experimental and computational studies are carried out on much simpler model membranes. Model membranes are hydrated lipid bilayers of a controlled lipid composition typical of the specific biomembrane. Lipid bilayers have three distinct regions, namely the bulk water region, the polar interface consisting of the lipid heads and water molecules, and the nonpolar bilayer core consisting of the lipid hydrocarbon chains. The interfacial region separates the other two regions and constitutes the first barrier preventing free movement of molecules across the bilayer. Moreover, many important processes occur there (Bondar and Lemieux, 2019). The interface thus plays an essential role in the functioning of the biomembrane.

Even in simple model membranes the lipid/water interface is structurally and dynamically complex. Structurally, because it consists of different types of polar, nonpolar and charged chemical groups and water molecules; dynamically, because the groups are in constant motion and the interfacial water molecules, even though predominantly bound to the lipid head groups (Markiewicz et al., 2015; Calero and Franzese, 2019), undergo rotational and translational motion and exchange with bulk water fast (Rog et al., 2009). Intermolecular interactions, dynamics and spatial organisation of the lipid head groups and water molecules at the interface are strongly interrelated, and this mutual dependence regulates the properties of the interface and thus of the membrane (Disalvo and Disalvo, 2015; Nickels et al., 2015; Frias and Disalvo, 2021).

The lipid composition of the matrix depends on the type of biomembrane within the cell and the function of the cell within the organism. The matrix of the mammalian plasma membrane consists primarily of glycerophospholipids (PL), i.e. phosphatidylcholine (PC), phosphatidylethanolamine (PE), phosphatidylserine (PS), and sphingomyelin (SM), with one saturated and the other mono-*cis*-unsaturated acyl chains (van Meer et al., 2008). PE together with phosphatidylglycerol are the main lipid representatives of the inner bacterial membranes (Dowhan, 1997). The main constituents of thylakoid membranes of chloroplasts are glycolipids with the galactose moieties and the glycerol backbone as the head group, i.e. monogalactosyldiglyceride (MGDG) and digalactosyldiglyceride (DGDG), and both α-linolenoyl (di-18:3, *cis*) acyl chains (Dormann and Benning, 2002). Poly-unsaturation of galactolipid acyl chains is indispensable for proper functioning of thylakoid membranes as summarised in Bratek et al., 2019 (Bratek et al., 2019) and citations therein. Lipopolysaccharides and lipid A are the main constituents of the outer membrane of Gram-negative (G^–^) bacteria (Brandenburg et al., 2016).

Due to the importance of the mammalian plasma membrane and the fact that they are relatively straightforward to handle, single or binary mixed PL bilayers as well as those also containing other natural membrane components have been extensively studied and much is known about their interfaces. The lipid/water interface of PL bilayers has been studied using experimental methods e.g. (Gawrisch et al., 1978; Volkov et al., 2007a; Volkov et al., 2007b; Disalvo et al., 2008; Zhao et al., 2008; Beranova et al., 2012; Cheng et al., 2014; Pokorna et al., 2014), although more detailed information about its properties has been provided by computer modelling, e.g. (Rog et al., 2009; Berkowitz and Vacha, 2012; Disalvo et al., 2014; Nickels et al., 2015; Pasenkiewicz-Gierula et al., 2016; Laage et al., 2017; Elola and Rodriguez, 2018; Martelli et al., 2018; Srivastava and Debnath, 2018; Tian and Chiu, 2018; Calero and Franzese, 2019; Kucerka et al., 2019; Srivastava et al., 2019; Deplazes et al., 2020; Luo et al., 2020; Szczelina et al., 2020; Hande and Chakrabarty, 2022).

In contrast to phospholipids, glycolipids are relatively less frequently studied in spite of their widespread occurrence. Publications on the lipid/water interface of galactolipid bilayers either in the lamellar or non-lamellar phases are rather scarce. Experimental studies were carried out on bilayers consisting of galactolipids with 18:3, 18:2 18:1, 18:0 and 16:0 acyl chains, e.g., (Shipley et al., 1973; Marra, 1986; Mcdaniel, 1988; Webb and Green, 1991; Bottier et al., 2007) whereas computational studies were carried out on di-18:3 MGDG, e.g., (Markiewicz et al., 2015; Baczynski et al., 2018; Szczelina et al., 2020), 80% di-18:3 DGDG and 20% 18:3–16:0 DGDG (Kanduc et al., 2017) and di-16:0 glucolipid and di-16:0 galactolipid (Rog et al., 2007) bilayers. Also publications on the interface of lipopolysaccharide and lipid A bilayers are not numerous, e.g. (Snyder et al., 1999; Wu et al., 2013; Murzyn and Pasenkiewicz-Gierula, 2015; Kim et al., 2016; Luna et al., 2021; Paracini et al., 2022).

Whereas di-18:3-*cis* DGDG is a bilayer-forming lipid (Deme et al., 2014), di-18:3-*cis* MGDG is not (Deme et al., 2014); due to the cone shape under ambient conditions it forms an inverse hexagonal (H_II_) phase in water spontaneously (Sanderson and Williams, 1992). In this study, the organisation of the lipid/water interface of di-18:3-*cis* MGDG bilayers in different lyotropic phases and of the di-18:3-*cis* DGDG lamellar bilayer are analysed and compared. In particular, lipid-water as well as direct and water-mediated lipid-lipid interactions are identified. These interactions take place within the same bilayer interface but also between lipids belonging to the interfaces of apposing leaflets when a new phase begins to form. The strength and branching of inter-lipid interactions at the DGDG bilayer interface are analysed using a formal network analysis approach. The analysis demonstrates that the interactions together with the lipid head groups form a dynamic but stable and extended network. The topological properties of the network are determined and compared with those of the MGDG bilayer (Szczelina et al., 2020).

## 2 Systems and methods

### 2.1 Simulation systems

**FIGURE 1:**
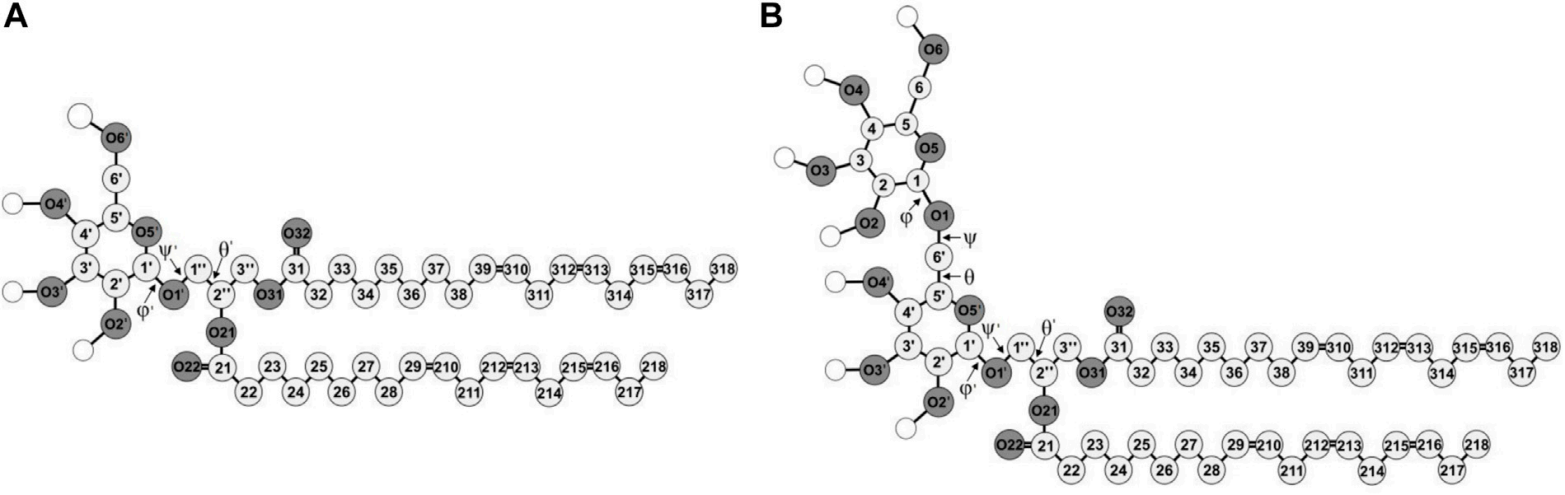
Molecular structures of di-18:3-*cis* **(A)** MGDG and **(B)** DGDG. The numbering of the acyl chains and glycerol backbone atoms is according to Sundaralingam’s nomenclature (Sundaralingam, 1972), with an exception for the C1″ and C3″ carbon atoms that are swapped here. The numbering of the galactose ring atoms is according to IUPAC convention (McNaught, 1996). The numbers of the carbon and oxygen atoms of the β ring are marked with ′ and the C1, C2 and C3 atoms of the glycerol backbone are marked with ″ to distinguish the atoms of the α and β galactose rings and of the glycerol. The chemical symbol for carbon atoms, C, is omitted and the hydrogen atoms are not shown except for the polar ones shown as empty circles. Oxygen (O) atoms are dark and the carbon atoms are light *grey* circles, respectively.

**FIGURE 2:**
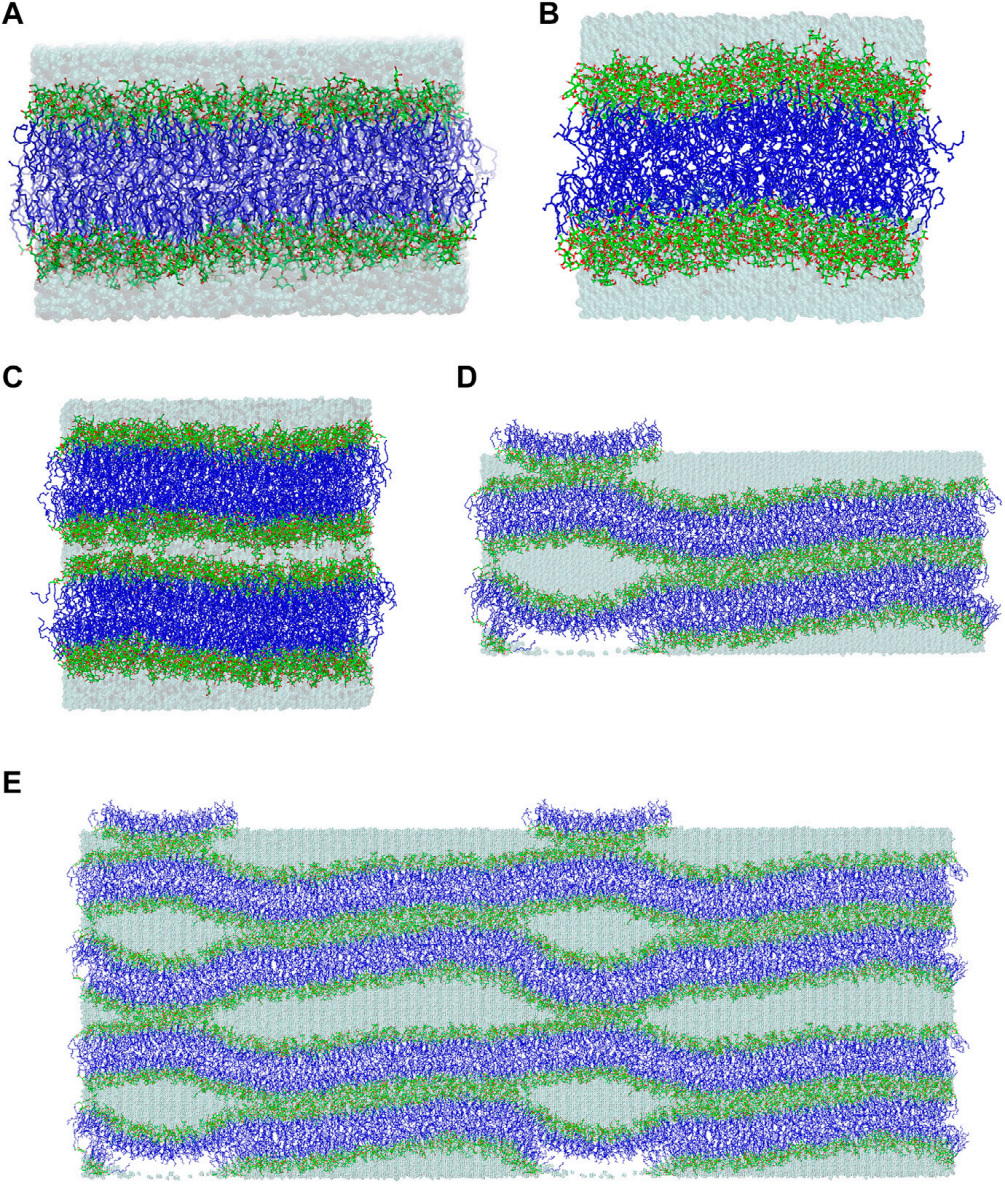
Final structures of the **(A)** MGDG and **(B)** DGDG bilayers after 320 and 1,050 ns of MD simulations, respectively. **(C)** Initial and **(D)** final (after ∼1,800 ns of MD simulation) structures of the double MGDG bilayer (W15). **(E)** The image of W15 in **(D)** was replicated along the *x*- and *z*-axis over periodic boundaries. The atoms are represented in standard colours, except for acyl chain carbon atoms, which are dark *blue*. The water is shown as a transparent *blue* surface. The hydrogen atoms are not shown.

In this molecular modelling study, the lipid/water interface of galactolipid systems in three different lyotropic liquid-crystalline phases is investigated. The galactolipids used to build the systems are monogalactosyldiglyceride (MGDG) and digalactosyldiglyceride (DGDG), each with both α-linolenoyl (di-18:3, *cis*) acyl chains (Figure 1). The investigated phases are MGDG and DGDG lamellar bilayers (Figure 2); the MGDG double bilayer, which forms the stalk phase (Figure 2); and the MGDG inverse hexagonal phase (H_II_) (Bratek et al., 2019).

The construction and conformational analysis of the computer model of the di-18:3-*cis* MGDG molecule (Figure 1) are described in Refs (Baczynski et al., 2015; Baczynski et al., 2018) and those of di-18:3-*cis* DGDG molecule (Figure 1) are described in Supporting Information (SI). MGDG and DGDG with poly-unsaturated acyl chains were chosen because of their widespread occurrence in Nature and the role they play in photosynthetic membranes (Dormann and Benning, 2002). MGDG and DGDG lamellar bilayers were built from scratch using the Packmol package (Martinez et al., 2009). The MGDG bilayer consisted of 450 MGDG molecules (15 × 15 in each leaflet); the DGDG bilayer consisted of 200 DGDG molecules (10 × 10 in each leaflet). The bilayers were hydrated with 30 H_2_O/lipid, i.e. with 13,500 and 6,000 H_2_O molecules, respectively, and MD simulated; the MGDG bilayer for 320 ns (Figure 2A), and the DGDG bilayer for 1,050 ns (Figure 2B). 30 H_2_O/lipid in the MGDG and DGDG bilayers is more than required for their full hydration. The hydration of DGDG in the lamellar phase and MGDG in hexagonal phase is similar (Brentel et al., 1985) and is 16 H_2_O/DGDG (Crowe et al., 1990) and ∼19 H_2_O/DGDG (Rand and Parsegian, 1989) and 13–14 H_2_O/MGDG (Brentel et al., 1985; Crowe et al., 1990; Selstam et al., 1990). This hydration corresponds to about 22% water by weight in both bilayers (Shipley et al., 1973).

The MGDG bilayer was validated in Ref. (Baczynski et al., 2015). Because a pure di-18:3-*cis* MGDG bilayer does not form in water spontaneously, e.g. (Dormann and Benning, 2002) there are no experimental data for this bilayer. Therefore, the MGDG bilayer was validated indirectly by comparing its structural properties with those of a pure di-16:0 MGDG bilayer (Rog et al., 2007; Lopez et al., 2013) and binary di-18:3 MGDG-DMPC bilayers (Kapla et al., 2012); also, with the help of a well-studied dioleoylPC (DOPC) bilayer, e.g., (Liu and Nagle, 2004; Kucerka et al., 2005; Kucerka et al., 2008). The DGDG bilayer is validated in the Results section.

A stalk is a crucial intermediate in the membrane fusion mechanism (Kozlovsky et al., 2004; Kasson and Pande, 2007). This is a local connection of lipids that belong to the inner leaflets of two bilayers which come into close contact (Figures 2D,E) as a result of their partial dehydration, and involves lipid mixing between these leaflets (Kozlov et al., 1989; Ohta-Iino et al., 2001; Salditt and Aeffner, 2016). The MGDG stalk was generated in MD simulation of the double bilayer system. The double bilayer was constructed by duplicating the MGDG bilayer after 300 ns of MD simulation and placing one bilayer on top of the other (Figure 2C and film Supplementary Video S1). The intra-bilayer water layer contained 6,750 H_2_O molecules (15 H_2_O/MGDG) and the outer water layer contained 13,500 H_2_O molecules (30 H_2_O/MGDG). The double bilayer (W15 system) was MD simulated for nearly 2,000 ns (Figure 2D). The first vertical connection between the head groups of two galactolipid molecules across the “inner” water layer formed spontaneously (Kozlovsky et al., 2004) within 1 ns of MD simulation (Supplementary Figure S3). A detailed description of the simulation and stalk formation will be presented elsewhere.

The construction of the MGDG H_II_ phase, its MD simulation and validation were described in detail in Ref. (Bratek et al., 2019). Several structures of the phase were tested before its stable computer model was achieved. The stable H_II_ phase consisted of sixteen cylinders, each containing 5,400 water molecules, which corresponded to 30 H_2_O/MGDG. The MGDG H_II_ phase generated in 3-µs MD simulation was analysed to obtain such basic structural parameters as hexagonal lattice constant, circular and effective radii of the water channel, surface area/MGDG, and order parameter profiles for the MGDG acyl chains (Bratek et al., 2019). To validate the computer model, experimental data for mainly di-18:3 MGDG (Shipley et al., 1973), mainly di-18:2 MGDG (Bottier et al., 2007), dioleoylPE (DOPE) (Rand and Fuller, 1994) and palmitoyloleoylPE (POPE) (Rappolt et al., 2003) H_II_ phases, were used. The comparison with experimental data was performed by extrapolating the linear dependence of the experimental parameters on the H_II_ phase hydration level, to a hydration level of 30 H_2_O/MGDG in the computer-generated H_II_ phase.

### 2.2 Simulation parameters and conditions

Force field parameters for the α-linolenic chain, and the glycerol moiety of the galactolipids, were taken directly from the all-atom optimised potentials for liquid simulations (OPLS-AA) force field associated with the software package GROMACS 5.05 (Abraham et al., 2015) and supplemented with the partial charges on the glycerol backbone of galactolipids from Ref. (Maciejewski et al., 2014).

The head group of MGDG comprises a single β-D-galactose and the glycerol backbone to which the galactose is attached by an O-glycosidic bond called here β-1′-1″ linkage (cf. Figure 1). The head group of DGDG comprises two galactose moieties, α-D-galactose and β-D-galactose linked by an O-glycosidic bond called here α-1-6 linkage (cf. Figure 1), also attached to the glycerol backbone by the β-1′-1″ linkage. For the galactose moieties of MGDG and DGDG, OPLS-AA parameters for carbohydrates (Damm et al., 1997) were used. The parameters for MGDG and other mono-glycoglycerolipids have been tested successfully in previous atomistic MD-simulation studies (Rog et al., 2007; Baczynski et al., 2015; Markiewicz et al., 2015; Baczynski et al., 2018; Bratek et al., 2019; Szczelina et al., 2020). The DGDG bilayer is validated in the Results section. For water, the transferable intermolecular potential three-point model (TIP3P) was used (Jorgensen et al., 1983).

MD simulations of the lamellar galactolipid bilayers were carried out in the *NPT* ensemble, under a pressure of 1 atm and at a temperature of 295 K (22°C) using the software package GROMACS (Abraham et al., 2015). To control the temperature and pressure, for the first 20 ns of MD simulation, the Berendsen thermostat and barostat (Berendsen et al., 1984) were used, and then the Nosé-Hoover (Nose, 1984; Hoover, 1985) and the Parrinello-Rahman (Parrinello and Rahman, 1981) methods were used, respectively. The relaxation time for the temperature was 0.6 ps and for the pressure 1.0 ps. The temperatures of the solute and solvent were controlled independently, and the pressure was controlled anisotropically.

The linear constraint solver (LINCS) algorithm (Hess et al., 1997) was used to preserve the length of any covalent bond with a hydrogen atom, and the time step was set to 2 fs. The van der Waals interactions were cut-off at 1.0 nm. The long-range electrostatic interactions were evaluated using the particle-mesh Ewald summation method with a β spline interpolation order of 5, and a direct sum tolerance of 10^–5^ (Essmann et al., 1995). For the real space, a cut-off of 1.0 nm, three-dimensional periodic boundary conditions (PBC), and the usual minimum image convention, were used (Essmann et al., 1995). The list of non-bonded pairs was updated every 5 time steps.

The W15 system was MD simulated at 295 K for 320 ns (film SF1, SI). Then, to speed up the process of the MGDG stalk structure formation, the following ∼1.4-µs simulation was carried out at 333 K (60°C) with the time step of 1.5 fs. After that, the temperature was gradually lowered to 295 K and after reaching this temperature, MD simulation was continued for 100 ns with a 2-fs time step. The temperature profile of this simulation is shown in Supplementary Figure S4. All other simulation parameters and conditions as well as the simulation programme were the same as in the case of the lamellar systems.

All trajectories analysed in this paper were recorded every 1 ps.

The MGDG H_II_ phase was generated in a 3-µs MD simulation, also at 295 K (22°C) and the trajectory was recorded every 2 ps (Bratek et al., 2019).

Visualisation of the results was done with the VMD 1.9.3 (Humphrey et al., 1996) and PyMOL 1.8.4 (DeLano, 2010) programmes.

### 2.3 Network analysis

The methodology used to analyse the interaction network at the bilayer interface is described in detail in Ref. (Szczelina et al., 2020). The basis of network analysis and the main weighted network parameters are summarised here only briefly. Mathematically, a network can be described and modelled by means of graph theory. In the following, the terms “network” and “graph” are interchangeable. Here, the objects of the graph are lipid molecules (centres-of-mass) in one bilayer leaflet (nodes) and intermolecular interactions between them are the graph edges. Consecutive pairs of nodes connected by edges form a path. A cluster is a set of interconnected nodes where each node has a path to all other nodes. A cluster size is the number of nodes which make a particular cluster. A graph is connected when it is made of only one cluster. The node degree is the number of edges connecting this node to other nodes. The node strength is determined by the number of individual interactions that account for each edge of the node, the average energy of each type of interaction and lifetime of the edge. A network bridge is an edge removing which disconnects the graph.

Network analysis was carried out using NetworkX (Hagberg et al., 2008), a Python language software package for creating, manipulating, and studying the structure, dynamics, and functions of complex networks. Network bridges were identified using a bridge-finding algorithm that employs the chain decompositions described in Ref. (Schmidt, 2013). The network at the DGDG bilayer was visualised using Cytoscape (Shannon et al., 2003).

## 3 Results

### 3.1 Systems equilibration and validation

**FIGURE 3:**
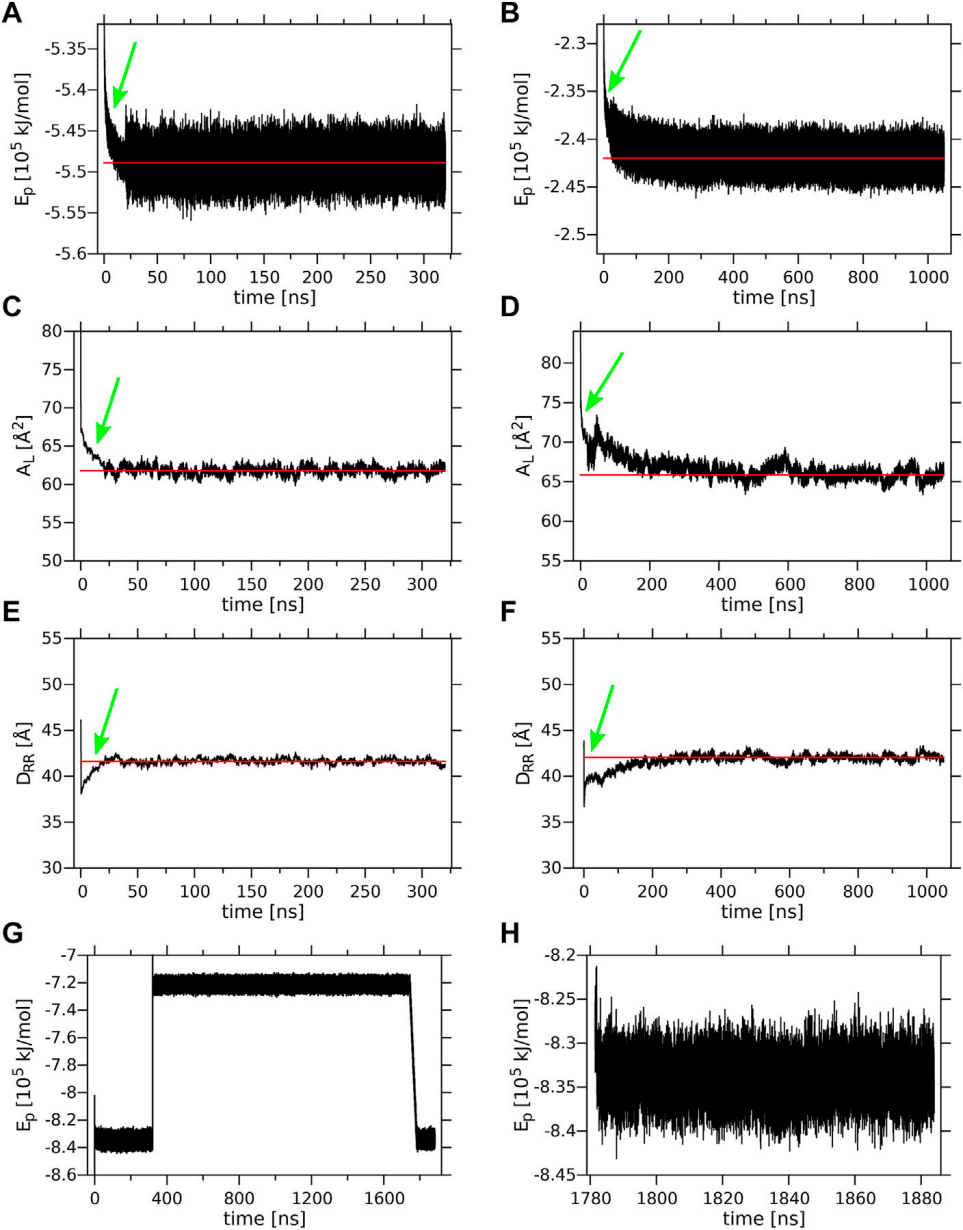
Equilibration of the lamellar MGDG and DGDG bilayers and the W15 system. Time profiles of the **(A)** MGDG and **(B)** DGDG potential energy (Ep); **(C)** MGDG and **(D)** DGDG average surface area per lipid (A_L_); **(E)** MGDG and **(F)** DGDG average bilayer width (D_RR_), during 320 and 1,050 ns, respectively, of MD simulations at 295 K. **(A–F)** The time (20 ns) when the *T* and *p* control methods were switched (cf. sec. 2.2) is marked with a *green* arrow; the *red* line shows the average value of a given parameter. Time profiles of Ep **(G)** for the whole ∼1,800-ns and **(H)** for the last 100-ns (when the system’s temperature was 295 K) of MD simulation of the W15 system.

Time profiles of the potential energy (Ep), the average surface area per lipid (A_L_) and the bilayer width (D_RR_) of the MGDG and DGDG bilayers are shown in Figure 3. The average A_L_ was obtained by dividing the simulation box surface area by the number of lipids in one bilayer leaflet. The average D_RR_ was defined and calculated as the distance between the average positions of the centres-of-mass of the single galactose rings (MGDG) or of the double galactose rings (DGDG), in the opposite bilayer leaflets, in a similar fashion to Ref. (Baczynski et al., 2015). Each of the three bilayer parameters converged to some constant value (Figure 3). The time profiles for the MGDG bilayer (Figures 3A,C,E) indicated that the bilayer equilibrated within ∼50 ns of MD simulation. However, the equilibration time of the DGDG bilayer was difficult to assess on the basis of the time profiles in Figure 3; therefore, the average values of Ep, A_L_ and D_RR_ were calculated at three time segments (400–500, 500–600, 600–1,050, Supplementary Table S2), which indicated that the DGDG bilayer equilibrated within ∼500 ns of MD simulation. The average values of Ep, A_L_ and D_RR_ for the MGDG and the DGDG bilayer are given in Table 1 and are marked in Figure 3 as straight *red* lines. Additionally, to compare with some experimental values, the average D_CC_ bilayer width was calculated as the distance between the average positions of the C2″ atoms (cf. Figure 1) in the opposite leaflets of the MGDG and DGDG bilayers (Table 1). For the MGDG bilayer the averages presented below were obtained for the time range 200–300 ns, while for the DGDG bilayer they were obtained for the range 900–1,000 ns of the respective MD simulations. Errors in the average values derived are standard deviation estimates.

**TABLE 1:** Mean values of the simulated systems parameters.

System	Ep [10^5^ kJ/mol]	A_L_ [Å^2^]	D_RR_ [Å]	D_CC_ [Å]	Tilt [°]
MGDG	−5.488 ± 0.02	61.77 ± 0.50	41.60 ± 0.28	34.59 ± 0.25	β: 32
DGDG	−2.420 ± 0.01	65.84 ± 0.63	42.04 ± 0.33	33.39 ± 0.31	α: 30 β: 36
W15	−8.340 ± 0.02	64.26 ± 0.72	40.39 ± 0.39	33.80 ± 0.40	β: 42

**FIGURE 4:**
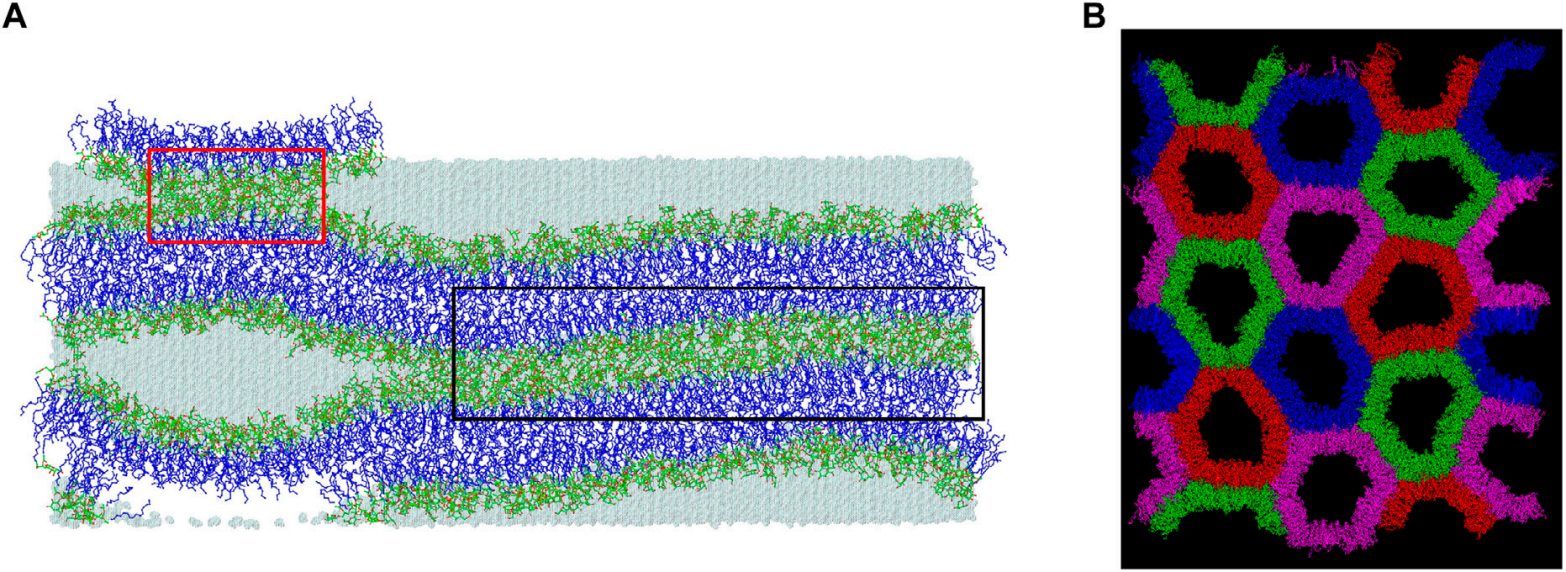
Three regions of W15 and top view of the H_II_ phase. **(A)** The concave region is not marked; the larger connect-15 region formed through the “inner” water layer (15 H_2_O/MGDG) is marked with a *black* frame and the smaller connect-30 region newly forming over the PBC through the “outer” water layer (30 H_2_O/MGDG) is marked with a *red* frame. The atoms are represented in standard colours, except for the acyl chain carbon atoms which are dark *blue*. The water is shown as a transparent *blue* surface. The hydrogen atoms are not shown. **(B)** Top view of the H_II_ phase generated Ref. (Bratek et al., 2019) after 3 µs of MD simulation. The water-filled cylinders are in different colours to show the H_II_ phase structure better. The water molecules are not shown.

Time average values of the potential energy (Ep), surface area per lipid (A_L_) and bilayer width (D_RR_, and D_CC_) (see text) as well as the preferred tilt angle (maximum of the ω angle probability distribution in Supplementary Figure S5) of the β and α rings for the MGDG, and DGDG, bilayers and the W15 system, MD, simulated at 295 K. The W15 system was cooled from 333 to 295 K and MD, simulated at this temperature for 100 ns (see Figure 3G); the average value of Ep was calculated for the whole W15 system and those for A_L_, D_RR_, and D_CC_, for its “flat” part (marked with a *black* frame in Figure 4A) over the last 60 ns. The errors are standard deviation estimates.

The equilibrated lamellar MGDG and DGDG bilayers are shown in Figures 2A,B, respectively.

The values for A_L_ of 61.77 ± 0.50 Å^2^ and D_RR_ of 41.60 ± 0.28 Å obtained in this study for the MGDG bilayer are very close to those obtained in Ref. (Baczynski et al., 2015), where the MGDG bilayer was validated (cf. sections 2.1).

The values for A_L_ of 65.84 ± 0.63 Å^2^ and D_RR_ of 42.04 ± 0.33 Å obtained in this study for the DGDG bilayer MD simulated for 1 µs can be compared with those published in the literature—for A_L_ they range between 63 and 78 Å^2^ and for D_RR_ between 41 and 44 Å. Most of the published values of A_L_ and D_RR_ were obtained from MD simulations, either coarse grained of 64 ± 1 Å^2^ and of 41 Å, respectively, for the di-16:0 DGDG bilayer (Lopez et al., 2013) and 63 ± 1 Å^2^ and 44 ± 3 Å, respectively, for the di-18:3 DGDG bilayer (Navarro-Retamal et al., 2018), atomistic united-atom of ∼78 Å^2^ and 41.7 Å, respectively, for the mixed DGDG bilayer containing 80% di-18:3 DGDG and 20% 18:3–16:0 (Kanduc et al., 2017) or atomistic all-atom of 67 Å^2^ and 42 Å, respectively, for the di-16:0 DGDG bilayer (Lopez et al., 2013). The experimental values of A_L_ and D_RR_ obtained using X-ray diffraction are ∼75 Å^2^ and 41.6 Å, respectively (Shipley et al., 1973), and of D_RR_ obtained using neutron diffraction is 41 Å (Mcdaniel, 1988), for the bilayers consisting of DGDG with 18:3, 16:0, 18:4, 18:2, and smaller amounts of 16:1, 18:0, 18:1 acyl chains, but mainly of di-18:3 DGDG (Shipley et al., 1973).

The results of MD simulations show some dependence of the acyl chain unsaturation on the bilayer structural properties, although it should be remembered that the computer models of the DGDG molecule used in those studies had different resolutions, thus the structural parameters derived may somewhat differ from one another. Other differences in the results may stem from the differing acyl chain compositions of the bilayers, e.g. (Shipley et al., 1973; Kanduc et al., 2017). Besides, the surface area/DGDG in the bilayer in Ref. (Kanduc et al., 2017), after initial equilibration, was kept constant, so its value may be not accurate.

The above comparisons demonstrate that the DGDG bilayer generated in this MD simulation study is effective in reproducing the basic bilayer properties determined in previous studies. Furthermore, the entries in Supplementary Table S1 imply that the conformational states of the DGDG head group concur well with experimental, e.g. (Hirotsu and Higuchi, 1976; Wormald et al., 2002; Ziolkowska et al., 2007; Nakae et al., 2018) and computer simulation, e.g. (Peric-Hassler et al., 2010) data for other disaccharides (cf. SI). Thus, the conclusion that the DGDG bilayer is positively validated is justified.

The initial and final structures of the W15 system are shown in Figures 2C,D. W15 is in the process of stalk phase formation (film Supplementary Video S1) and thus is not at equilibrium. Nevertheless, its energy profile (Figure 3G) was calculated for the whole MD simulation time of ∼1,800 nsas well as for the last 100 ns (Figure 3H), when the temperature, after lowering from 333 to 295 K, was stable at 295 K (cf. Methods). The average value of the whole system’s Ep, as well as the values of A_L_, D_RR_ and D_CC_ for its “flat” part (marked with a *black* frame in Figure 4A) calculated over the last 60 ns of MD simulation of the W15 systems equilibrated at 295 K, are given in Table 1.

### 3.2 Lipid-water H-bonds

MGDG has four OH groups that are both donors and acceptors of hydrogen bonds (H-bond) and six O atoms that are only acceptors of H-bonds (Figure 1) can thus make numerous H-bonds with water molecules. The average numbers of particular types of the MGDG-water interactions are given in Table 2.

**TABLE 2:** Number of lipid-water interactions in the MGDG and DGDG lamellar bilayers.

Bilayer	MGDG	DGDG
# H-bonds/head	9.37 ± 0.10	12.83 ± 0.19
# H-bonds/rings (H; O)	6.34 ± 0.08 (2.54 ± 0.05; 3.81 ± 0.05)	9.95 ± 0.17 (3.88 ± 0.08; 6.06 ± 0.12)
# H-bonds/α ring	–	5.81 ± 0.12
# H-bonds/β ring	–	4.14 ± 0.10
# H-bonds/gly	3.02 ± 0.06	2.88 ± 0.06
# H-bonded H_2_O/head	7.14 ± 0.09	9.07 ± 0.15
# WB/head	1.70 ± 0.06	2.89 ± 0.15
# ring-ring WB/head	0.80 ± 0.04	1.81 ± 0.12
#α ring-α ring WB/head	–	0.59 ± 0.06
#α ring-β ring WB/head	–	0.82 ± 0.07
#β ring-β ring WB/head	–	0.40 ± 0.05
# gly-gly WB/head	0.26 ± 0.02	0.24 ± 0.03
# gly-ring WB/head	0.63 ± 0.04	0.84 ± 0.06

Average numbers of lipid-water H-bonds (# H-bonds); H-bonded water molecules (#H bonded H_2_O) and water bridges (# WB) per lipid head, rings and glycerol backbone (gly) and additionally per α and β rings of DGDG, at the interface of the MGDG, and DGDG, bilayers. In parenthesis are the numbers of interactions via H (H-bond donor) and O (H-bond acceptor) atoms of the ring moieties. The glycerol backbone includes the O1’ atom (cf. Figure 1).

DGDG has seven OH groups that are both donors and acceptors of H-bonds and eight O atoms that are only acceptors of H-bonds (Figure 1). Accordingly, the average numbers of DGDG-water interactions (Table 2) are greater than those of MGDG, although somewhat smaller than expected.

Water molecules bind preferentially to the MGDG and DGDG rings and are 50% more often H-bond donors than acceptors (Table 2). In the water-glycerol H-bonding, water is the only H-bond donor. The number of water-glycerol H-bonds is only slightly smaller in the DGDG than the MGDG bilayer (Table 2).

The smaller than expected number of H-bonds with water and H-bonded water molecules in the DGDG than the MGDG bilayer is to some extent compensated by the larger number of water bridges (WB) (Pasenkiewicz-Gierula et al., 1997), which link pairs of galactolipid head groups. WBs form predominantly between galactolipid rings, although the number of glycerol-ring WBs in both bilayers is also quite significant (Table 2).

From Figures 2D, 4A it is apparent that the W15 system has two distinct regions—“region of full hydration” and “region of reduced hydration” (Figure 4A). The “full hydration region”, which is called concave is not marked in Figure 4A. The larger “reduced hydration” region forms as a result of the local cross-water connection of two inner leaflets of the double bilayer that were originally separated by the thinner “inner” water layer (15 H_2_O/MGDG). It is marked with a *black* frame in Figures 4A and is called connect-15. The smaller “reduced hydration” region is still forming over the PBC as a result of the local cross-water connection of two outer leaflets of the double bilayer that were originally separated by the thicker “outer” water layer (30 H_2_O/MGDG). It is marked with a *red* frame in Figures 4A and is called connect-30. The connections are more visible in Figure 2E.

The average number of each type of MGDG-water interaction in a specified region of the W15 system and in the MGDG H_II_ channels is given in Table 3. The numbers of MGDG-water H-bonds and water bridges (horizontal) as well as H_2_O molecules H-bonded by MGDG in the concave region of W15 are very similar to those in the MGDG lamellar bilayer, but those in the connect regions, particularly in the connect-15, are smaller (Tables 2, 3). The numbers of H-bonds and H-bonded H_2_O molecules in the MGDG H_II_ phase are smaller than those in the concave region, but are similar to those in the connect regions of W15 (Table 3).

**TABLE 3:** Number of lipid-water interactions in the MGDG lamellar and non-lamellar systems.

System	#H_2_O-lipid H-bond/head	#H Bonded H_2_O/head	WB/head horizontal	WB/head vertical	WB/head total
MGDG bilayer	9.37 ± 0.10	7.14 ± 0.09	1.70 ± 0.06	–	–
W15; concave	9.18 ± 0.18	6.99 ± 0.15	1.69 ± 0.10	–	–
W15; connect-15	8.13 ± 0.14	5.51 ± 0.07	1.57 ± 0.09	0.72 ± 0.06	2.28 ± 0.11
W15; connect-30	8.64 ± 0.23	6.25 ± 0.23	1.41 ± 0.16	0.56 ± 0.11	1.97 ± 0.20
H_II_	8.23 ± 0.04	6.01 ± 0.03	1.90 ± 0.03	–	–

Concave is the region of W15 that contains excess water (not marked in Figure 4A); connect-15, and connect-30 (cf. Main text) are regions of W15 that are marked with *black* and *red* frames, respectively, in Figure 4A. The inverse hexagonal MGDG, phase (H_II_) was generated in Ref. (Bratek et al., 2019). In the connect-15, and connect-30 regions, the numbers of the horizontal and vertical WBs (see text) are given. To make comparison easier, some data for the MGDG, bilayer from Table 2 were added to Table 3.

**FIGURE 5:**
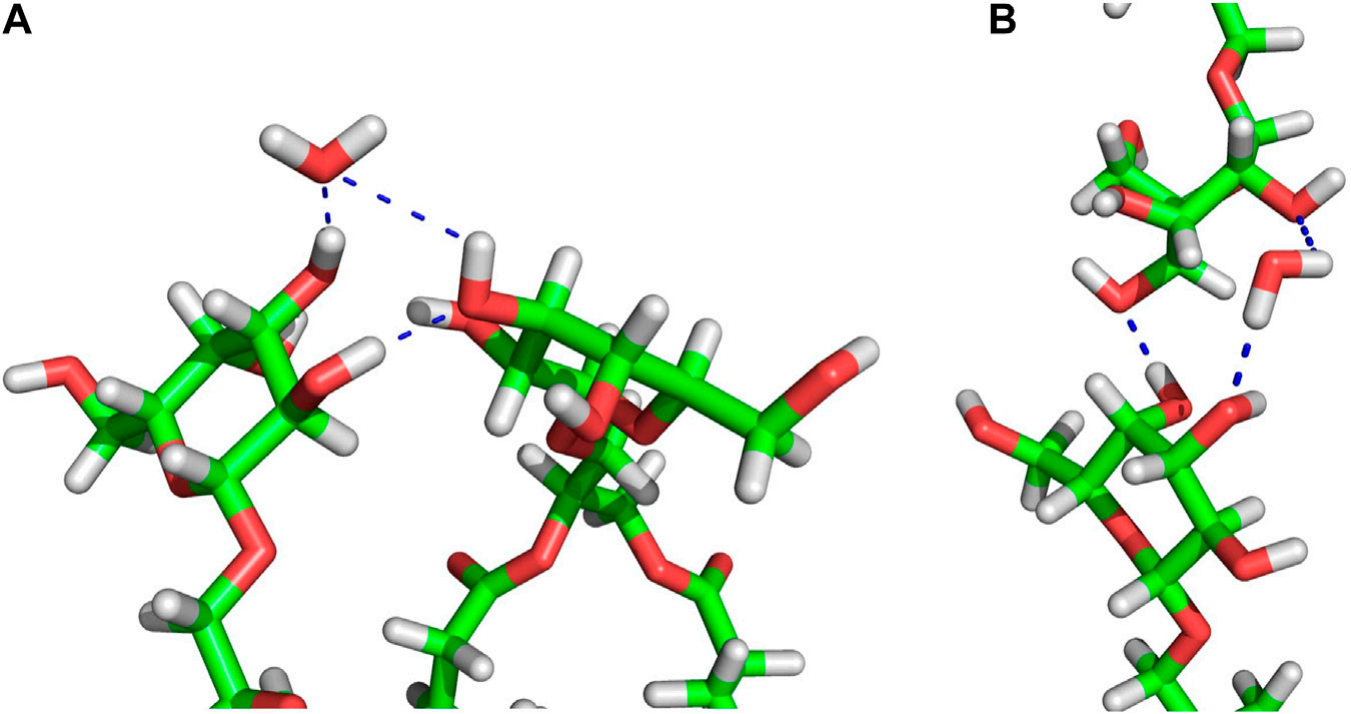
Examples of MGDG-MGDG H-bonds and water bridges at the interface of the W15 connect-15 region. **(A)** Horizontal (between lipids belonging to the same bilayer leaflet) interactions; **(B)** vertical (between lipids belonging to the apposing leaflets) interactions. The molecules are shown as sticks in standard colours (acyl chains are cut off). The dotted *blue* lines represent intermolecular interactions. In **(A)** the water molecule is an acceptor of two H-bonds, in **(B)** the water molecule is a donor of two H-bonds.

In the connect-15 and connect-30 regions, the horizontal and vertical WBs can be distinguished. The horizontal WBs are between lipids of the same bilayer leaflet (Figure 5A) and the vertical are between lipids of the apposing leaflets (Figure 5B). In both regions, the number of horizontal WBs is larger than that of the vertical ones but somewhat smaller than the number of them in the MGDG lamellar bilayer and the concave region. This could indicate that there is some competition between the horizontal and vertical WBs. Nevertheless, the total number of WBs (horizontal and vertical) in each connect region is larger than the number of those in the concave region and the lamellar bilayer. The number of water bridges (horizontal) in the MGDG H_II_ phase is higher than in the MGDG bilayer and any W15 region (Table 3).

### 3.3 Lipid-lipid interactions

The MGDG and DGDG heads have both H-bond acceptor and donor groups. Therefore, they can be linked by direct inter-lipid H-bonds at the lipid/water interface, in addition to water bridges, which are water-mediated lipid-lipid interactions (Table 2, 3). The numbers of direct H-bonds in the MGDG and DGDG lamellar bilayers, the W15 system and its specified regions, as well as in the MGDG H_II_ phase, are given in Table 4. The number of H-bond acceptor and donor groups of DGDG is 50% larger than that of MGDG; however, the number of DGDG-water H-bonds is only ∼37% greater, whereas the numbers of WBs and direct H-bonds are 70 and ∼130%, respectively, greater than those of MGDG (Tables 2, 4). This indicates that at the bilayer interface the head groups of DGDG interact preferentially with one another, rather than with water, whereas interactions between the MGDG head groups and water are relatively numerous.

**TABLE 4:** Number of lipid-lipid interactions.

System	# Head-head H-bonds/head	# Head-head WB/head
MGDG bilayer	0.87 ± 0.04	1.70 ± 0.06
#β ring-β ring/MGDG	0.57 ± 0.03	0.80 ± 0.04
DGDG bilayer	1.97 ± 0.08	2.89 ± 0.15
#α ring-α ring/DGDG	0.46 ± 0.03	0.59 ± 0.06
#α ring-β ring/DGDG	0.32 ± 0.03	0.82 ± 0.07
#β ring-β ring/DGDG	0.69 ± 0.07	0.40 ± 0.05
W15; concave	0.94 ± 0.06	1.69 ± 0.10
W15; connect-15	H: 0.90 ± 0.05	H: 1.57 ± 0.09
	V: 0.41 ± 0.04	V: 0.72 ± 0.06
	T: 1.31 ± 0.07	T: 2.28 ± 0.11
W15; connect-30	H: 0.81 ± 0.09	H: 1.41 ± 0.16
	V: 0.31 ± 0.16	V: 0.56 ± 0.11
	T: 1.12 ± 0.12	T: 1.97 ± 0.20
H_II_	1.33 ± 0.02	1.90 ± 0.03

Average numbers of direct lipid-lipid H-bonds (second column) in the MGDG, and DGDG, lamellar bilayers per head and per the α and β rings of DGDG; the concave, connect-15, and connect-30 regions of W15 (cf. Figure 4A) as well as in the inverse hexagonal MGDG, phase (H_II_). For comparison, the average numbers of WBs, from Table 2 and 3 are also given (third column). H, V and T stand for the horizontal, vertical and total direct H-bonds and WBs, respectively (see text).

To obtain a better insight, the numbers of ring-water and ring-ring interactions in the DGDG bilayer were calculated for the α and β galactose rings separately. The results given in Tables 2, 4 show that the number of intermolecular interactions of each DGDG ring is different.

In the connect regions of W15, the MGDG head groups form both horizontal and vertical inter-lipid H-bonds (Figure 5), as in the case of WBs. The average numbers of horizontal H-bonds in the three regions of W15 are similar to each other and also similar to the number of them in the MGDG lamellar bilayer. This implies that, in contrast to WBs, different H-bond donor and acceptor groups of MGDG are involved in formation of the horizontal and the vertical direct H-bonds.

The number of lipid-lipid H-bonds in the MGDG H_II_ phase is higher than in the MGDG bilayer or any W15 region (Table 4), as is the number of WBs.

### 3.4 Orientation of the galactolipid head group

The orientation of the MGDG head group in the bilayer is determined here, as in Ref. (Baczynski et al., 2018), by angle ω between the MGDG head group vector, which connects the C2″ atom in the glycerol and the O4’ atom in the galactose ring (Figure 1A), and the bilayer normal. Ref. (Baczynski et al., 2018) correlations between angle ω and the conformation of the torsion angles of the glycosidic linkages, and also between angle ω and the numbers of head-water and head-head interactions at the MGDG bilayer interface, were calculated. Significant conclusions of those calculations were that there was virtually no correlation between the orientation of the MGDG head group and the conformation of its glycosidic linkage, and that there was only a weak correlation between the MGDG head group orientation and the number of intermolecular interactions of the head.

The most probable (preferred) orientation, called here tilt, of the head group is angle ω, for which the ω distribution has the main maximum. The distributions of ω and tilts for MGDG in the bilayer and in the connect-15 region of W15 obtained in this study are shown in Supplementary Figure S5A, B and are given in Table 1, respectively. In the MGDG bilayer the ω distribution is smooth and the vector tilt is 32°. Both are similar to those in Ref. (Baczynski et al., 2018). In the connect-15 region the ω distribution has a long tail and the vector tilt is 42°. These results can possibly be linked to the somewhat uneven surface of the connect-15 region (Figure 4A) and to the onset of the rotation of some of the MGDG molecules in the process of formation of the stalk structure.

The tilt of the DGDG α ring in the bilayer was obtained from the distribution of angle ω between the α ring vector (C2″-O4 vector, Figure 1B) and the bilayer normal (Supplementary Figure S5C), and that of the DGDG β ring was obtained from the distribution of the ω angle between the β ring vector (C2″-O4’ vector, Figure 1B) and the bilayer normal (Supplementary Figure S5D); both tilts are given in Table 1. The tilt of the DGDG α ring of 30° is almost the same as that of the MGDG β ring of 32°. However, their ω distributions differ. In addition to the main maximum in the ω distribution of the α ring vector at 30°, there are smaller but clear maxima at ∼60°, ∼80° and the last one at ∼140°. These maxima indicate that the α ring may have three additional less populated but stable orientations.

The tilt of the DGDG β ring is 36°. Even though the tilts of the DGDG α and β ring vectors are similar, the rings belong to different planes (Supplementary Figure S6). The distribution of the angle between the planes of the α and β rings shown in Supplementary Figure S7 has two maxima. The higher, relatively narrow maximum is at 82° and the significantly lower one is at 162°. The angles are most likely determined by the preferred populations of the torsion angles of the α-1-6 and β-1′-1″ glycosidic linkages. On the basis of the results of Ref. (Baczynski et al., 2018) it is justified to assume the angles between the α and β ring planes do not depend on the α and β ring tilts.

### 3.5 Density profile of the terminal CH_3_ groups of galactolipid acyl chains

The density profiles of the terminal CH_3_ groups of the poly-*cis*-unsaturated α-linolenoyl acyl chains of MGDG and DGDG across the bilayer were calculated to estimate the probability of finding the groups in the interfacial region of each bilayer, and to compare this probability with the results of previous experimental, e.g. (Feix et al., 1984; Mihailescu et al., 2011) and computer simulation, e.g. (Mihailescu et al., 2011) studies. The profiles across the MGDG and DGDG bilayers and connect-15 region are shown in Supplementary Figure S8. The probability was calculated for each leaflet of the bilayers from the area under the fragment of the CH_3_ profile where the electron density of water is non-zero (Supplementary Figure S8). The estimated probability (averaged over both leaflets) of finding a CH_3_ group at the bilayer interface is ∼15% in the MGDG bilayer and ∼16% in the DGDG bilayer.

### 3.6 Network analysis of the DGDG bilayer

**FIGURE 6:**
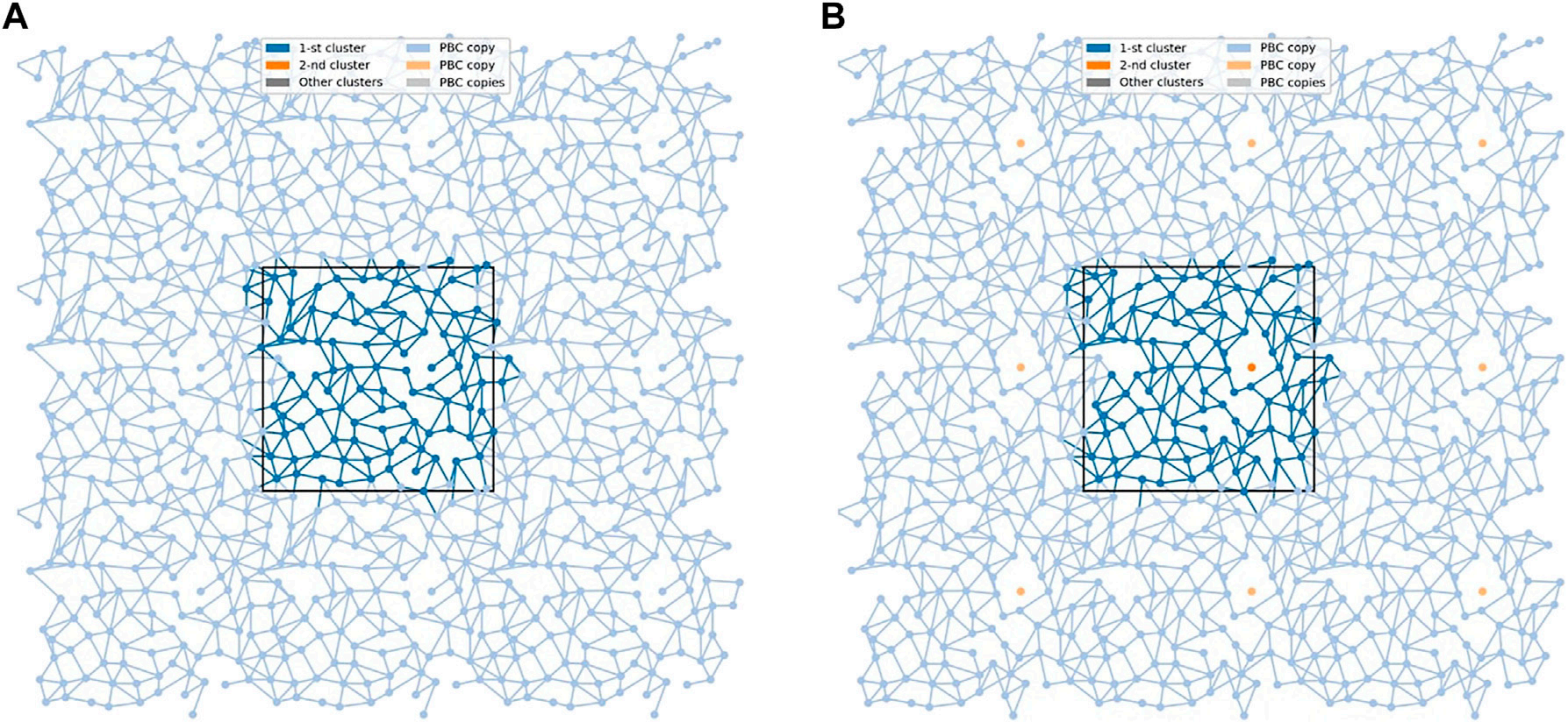
The networks of lipid interconnections at the interfaces of the DGDG bilayer at two time frames 1 ps apart. **(A)** The network is connected and **(B)** the connected network is broken into two clusters. The *black* rectangle depicts the basic simulation box. Nodes (centres-of-mass of the lipids) are presented as dots in the *x,y*-plane, and edges as lines connecting respective nodes. The largest cluster is in *blue* and a single-node cluster is in *orange*. To avoid problems with edges crossing PBC, 9 copies of each node are presented (strong colour tone for the cluster, soft colour tones for its 8 PBC copies); the edges are drawn in the basic simulation box and all its copies.

At the bilayer interface, the galactolipid head groups and interactions (H-bonds and water bridges) between them create a network of interactions. In Ref. (Szczelina et al., 2020) several topological properties of the network in the MGDG bilayers were determined. Here, the same methodology (cf. section 2.3) is used to analyse the interaction network in the DGDG bilayer. The values of the network parameters (cf. section 2.3), averaged over the last 50 ns of MD simulation of the DGDG bilayer, are given in Table 5, together with those obtained for the MGDG bilayers in Ref. (Szczelina et al., 2020). As the interacting groups of MGDG and DGDG head groups are the same, in this calculation the average energies of H-bonding and water bridging of DGDG are assumed to be the same as those of MGDG calculated in Ref. (Szczelina et al., 2020). The interaction network in the DGDG bilayer is presented in Figure 6 and its time changes are shown in film Supplementary Video S2.

**TABLE 5:** Mean values of the network parameters.

Bilayer (# lipids in a leaflet)	MGDG* (8 × 8)	4 MGDG* (16 × 16)	DGDG (10 × 10)
# H-bonds	1.04 ± 0.08	1.03 ± 0.04	1.90 ± 0.06
# water bridges	1.74 ± 0.13	1.69 ± 0.06	3.11 ± 0.12
# clusters	1.52 ± 0.53	3.27 ± 1.09	1.01 ± 0.06
size smallest	39.05 ± 21.78	28.83 ± 56.23	99.30 ± 5.86
size largest (%)	63.25 ± 0.99 (98.8)	252.80 ± 2.02 (98.7)	99.99 ± 0.06 (100)
# network bridges	5.12 ± 2.19	21.11 ± 4.44	0.21 ± 0.33
node strength	35.25 ± 2.03	34.86 ± 0.95	34.47 ± 1.09
Edge lifetime [ps]			
Direct H-bonds	1.73 ± 0.006	1.73 ± 0.004	0.86 ± 0.003
Water bridges	1.44 ± 0.007	1.43 ± 0.005	0.80 ± 0.004
Interaction energy [kcal/mol]			
Direct H-bonds	−5.12 ± 2.75	−5.12 ± 2.75	−5.12 ± 2.75
Water bridges	−14.23 ± 7.80	−14.23 ± 7.80	−14.23 ± 7.80

Average numbers (#) of lipid-lipid H-bonds and water bridges; average number of clusters (# clusters); average size of the smallest (size smallest) and largest (size largest) clusters (in parenthesis, % of the lipid molecules in one bilayer leaflet); average number of network bridges (# network bridges); average node strength; average lifetimes of inter-node edges (Edge lifetime) in networks via H-bonds (Direct H-bond) and via water bridges; average energy of the H-bond (Direct H-bond) and the water bridge interaction (Interaction energy) for the MGDG*, 4 MGDG* (Szczelina et al., 2020) and DGDG, bilayers MD, simulated at 295 K. The numbers for the DGDG bilayer are averages over the last 50 ns of the 1,050-ns MD simulation. The errors are standard deviation estimates, except for the errors in edge lifetimes, which are estimated as in Ref. (Szczelina et al., 2020).

The values given in Table 5 are graphically presented in Supplementary Figures S9,S10. These figures also provide additional information. The distribution of the node degrees shown in Supplementary Figure S9A indicates that the most probable degree in the DGDG bilayer is 5. This means that two DGDG head groups are most often connected by five individual inter-lipid interactions, and the smallest number of such interactions is two. The results shown in Supplementary Figure S9C demonstrate that only clusters of sizes 1, 2, 98, 99 and 100 have non-zero probability of forming and the probability of forming a cluster of size 100 is at least two orders of magnitude larger than that of the remaining ones. Time profiles of the average number of clusters and the average sizes of the smallest and the largest clusters are shown in Supplementary Figure S10; the averages are over two networks, each in one bilayer leaflet. Supplementary Figure S10A reveals that in each bilayer leaflet the network is connected for most of the time. However, from time to time one of the connected networks breaks for a short while into two clusters and the average number of clusters is then 1.5. Only in one case does the network break into three clusters (of sizes 1, 1 and 98) and the average number of clusters is then 2 (Supplementary Figure S10A). The average size of the smallest cluster is either 100 or nearly 50 (Supplementary Figure S10B) and that of the largest is either 100 or nearly 100 (Supplementary Figure S10C). For most of the time, the sizes of the smallest and the largest clusters are 100. The time profile of the average node strength plotted in Supplementary Figure S10D only fluctuates around the average value, and this indicates that the average node strength is stable during the simulation time.

## 4 Discussion

### 4.1 MGDG and DGDG bilayers

An interesting result of this study is that the DGDG bilayer equilibrated after a much longer time than the MGDG bilayer. This effect was previously noticed by Kanduč et al. (Kanduc et al., 2017) and explained as arising from the “pronounced hydrogen-bonding capabilities” of DGDG (Kanduc et al., 2017). Our results indicate that direct DGDG-DGDG H-bonds as well as WBs at the DGDG bilayer interface are indeed numerous (Table 4). The detailed spatial organisation of these inter-lipid links is revealed by network analysis (Hagberg et al., 2008) (cf. Section 3.6). The network of the connections (H-bonds and WBs) is shown in Figure 6 and its dynamics in film Supplementary Video S2. The figure, the film and the network parameters in Table 5 as well as Supplementary Figures S9,S10 demonstrate that the connections are not only numerous, but also extended, and branched and for most of the time encompass all DGDG molecules in each bilayer leaflet.

The interaction network in the DGDG bilayer is qualitatively similar to those in the MGDG bilayers (Szczelina et al., 2020), but is considerably more stable. It also differs in some other aspects. The most probable node degree in the DGDG bilayer is 5 (Supplementary Figure S9A), whereas in the MGDG bilayer it is 3 (Szczelina et al., 2020). The average number of clusters is ∼1 (Table 5; Supplementary Figures S9B,S10A), whereas in the MGDG bilayer, depending on its size, it is ∼1.5 or ∼3 (Table 5). The number of network bridges (0.21) is much smaller than in the MGDG bilayers (Table 5). The small number of network bridges in the DGDG bilayer indicates that the connected network disconnects rarely (Supplementary Figure S10A) and much less often than in the 4 MGDG bilayer, cf. Supporting Information of Ref. (Szczelina et al., 2020).

Probably due to the greater A_L_ (65.8 vs. 61.8 Å^2^), the lifetimes of network edges consisting of only H-bond interactions and of only water bridge interactions in the DGDG bilayer are about half the length of those in the MGDG bilayers (Table 5). However, because the numbers of individual inter-lipid interactions are about twice as great and the energies of the interactions are the same, the node strength in the DGDG and MGDG bilayers are similar.

In the DGDG bilayer, as in the MGDG bilayers (Szczelina et al., 2020), the average node strength is stable during the analysis time (Supplementary Figure S10D). This indicates that the pattern of lipid interconnections at the bilayer interface is also stable in spite of the relatively short lifetimes of the network edges (Table 5), their fast rearrangements (film Supplementary Video S2) and the overall dynamics of the bilayer interfacial region.

Despite the fact that the network of interconnections at the DGDG bilayer interface is more stable, extended and branched than that at the MGDG bilayer interface, the large head group and cylindrical shape of the DGDG molecule prevent formation of non-bilayer phases, as is the case with the MGDG aggregates.

An apparent disproportion in the number of inter-lipid links at the bilayer interface between the DGDG and the MGDG bilayer (cf. section 3.3) can possibly be explained as follows. The tilt of the MGDG β galactose rings relative to the bilayer normal is 32° (Table 1), thus their polar groups are quite exposed to the water phase. In contrast, the DGDG β rings are screened from water by the α rings so they make fewer H-bonds with water than those of MGDG (Table 2). Due to smaller hydration, the polar groups of the DGDG β ring make fewer ring-ring WBs but more direct ring-ring H-bonds than those of the MGDG β ring and the DGDG α ring (Table 4). As a consequence of the hydration disparity of the DGDG α and β rings, the β-α WBs are more numerous and the β-α H-bonds are less numerous than those of the α-α rings (Tables 2, 4).

The tilt of the DGDG α galactose rings (O4-C2″ vector) is 30° and is practically the same as that of the MGDG β galactose rings, which is 32° (Table 1). However, the average numbers of ring-water H-bonds and ring-ring H-bonds and WBs made by the DGDG α ring are smaller than those made by the MGDG β ring (Tables 2, 4). This is because in addition to the α ring-water and α-α rings interactions, the α ring also interacts with the DGDG β ring. These interactions can be seen in the ω angle distribution in Supplementary Figure S5 as secondary maxima. They indicate that the DGDG α ring, on top of the preferred one, has three other less populated but stable orientations. Two of these orientations (∼60° and ∼80°) are possibly stabilised by its interactions with the β ring (particularly WBs) and one (∼140°) with the glycerol backbone (Tables 2, 4). The values obtained for the DGDG α ring orientation are only in partial agreement with conclusions derived on the basis of experimental data that “the polar head group of this lipid (DGDG) is oriented parallel to the plane of the bilayer” (Marra, 1986; Mcdaniel, 1988). The distribution of angle ω in Supplementary Figure S5 indicates that only a small fraction of the DGDG α rings is oriented parallel to the bilayer plane.

Previous experimental, e.g. (Feix et al., 1984; Mihailescu et al., 2011) and computer simulation, e.g. (Mihailescu et al., 2011) studies have revealed that the terminal CH_3_ groups of PL acyl chains can locate in the bilayer interfacial region. Using the electron-electron double-resonance methodology Felix et al. (Feix et al., 1984) showed that the probability of finding the CH_3_ group of a saturated acyl chain at the interface is 14%. While using the neutron diffraction methodology Mihailescu et al. (Mihailescu et al., 2011) showed that the probability of finding the CH_3_ group of a mono-*cis*-unsaturated acyl chain at the bilayer interface is 20%. This location of the CH_3_ groups was also found in an MD simulation study (Mihailescu et al., 2011). In this study the probability of finding the CH_3_ group of a poly-*cis*-unsaturated acyl chain was calculated from the electron densities of the CH_3_ groups and the water of each bilayer leaflet (Supplementary Figure S8), and was found to be ∼15% in the MGDG bilayer and ∼16% in the DGDG bilayer. Thus, these results predict that the terminal CH_3_ group not only of a saturated and a mono-unsaturated acyl chain of PC but also of a poly-unsaturated acyl chain of MGDG and DGDG can be found in the interfacial bilayer region.

### 4.2 W15 system

In the W15 system, two MGDG bilayers were initially placed parallel to each other and separated by two water layers, namely, the thinner “inner” water layer containing 6,750 H_2_O molecules (15 H_2_O/MGDG) and the thicker “outer” water layer containing 13,500 H_2_O molecules (30 H_2_O/MGDG) (Figure 2). In the course of MD simulation, the distance between the bilayers decreased in some places and increased in some others, indicating the onset of stalk structure formation (film Supplementary Video S1). The first vertical lipid-lipid contact across the “inner” water layer formed within the initial 1 ns of MD simulation at 295 K (Supplementary Figure S3). Local partial dehydration leading to formation of the connect-15 region can be seen in film SF1 (SI). In this dehydration process, each MGDG molecule loses on average approximately one H-bond with water and ∼1.5 H-bonded water molecules, but gains ∼0.6 WBs and ∼0.4 inter-lipid H-bonds (Table 3). Thus, interactions with water are replaced by lipid-lipid interaction. Water molecules move from the connect to the concave regions; this process is relatively fast as can be deduced from film Supplementary Video S1.

Hydration of MGDG molecules as well as the number of inter-lipid interactions in the W15 concave region are practically the same as in the MGDG bilayer. This might be because the W15 is in the process of stalk structure formation. The stalk structure involves lipid mixing between apposing leaflets (Kozlov et al., 1989; Ohta-Iino et al., 2001; Salditt and Aeffner, 2016), which requires rotation of lipid molecules so as to transform the concave region into a water-filled tube, whose inner surface consists of lipid heads. This rotation only started in the connect-15 region of W15, so the properties of the concave region are more like those of the MGDG bilayer than of the MGDG H_II_ phase. The onset of rotation of some of the MGDG molecules in W15 can be deduced from the long tail in the ω distribution for the MGDG ring vector in the connect-15 region (Supplementary Figure S5B), and the relatively high value of the MGDG tilt, being 42° (Table 1).

### 4.3 H_II_ phase

Details of the construction and MD simulation of the MGDG H_II_ phase are described in Ref. (Bratek et al., 2019). The MGDG H_II_ phase consisted of sixteen cylinders (Figure 4B), each filled with 5,400 water molecules (30 H_2_O/MGDG). In that paper, the basic structural parameters of the H_II_ phase were identified (cf. section 2.1). In this paper, the average hydration and the average number of inter-lipid interactions of the MGDG head groups are calculated. Compared to the MGDG bilayer and the concave region of W15, the MGDG head groups in the H_II_ phase are less hydrated than in the bilayer, but their hydration is similar to that in the connect regions of W15, whereas their mutual interactions are more numerous than in the bilayer and W15.

### 4.4 Effect of acyl chains on the H_II_ phase structure

Combined X-ray, neutron scattering and MD simulation studies indicate that the length and mono- and poly-unsaturation of PL acyl chains have an impact, among others, on the lipid surface area in PC, e.g. (Pabst et al., 2010; Marquardt et al., 2020) and PE, e.g. (Kucerka et al., 2015) bilayers. Using a different experimental approach, the effect of the length and unsaturation of the acyl chains on lipid hydration in PC and PE monolayers is revealed (Maltseva et al., 2022). Yet, our previous MD simulation study on *cis*- and *trans*-mono-unsaturated PC bilayers indicates that the conformation of the double bond does not have much impact on the lipid surface area (Murzyn et al., 2001).

The results for the H_II_ phase are in contrast with those for lamellar PL bilayers. The experimentally derived structural parameters such as hexagonal lattice constant (*dhex*) and radius of the water channel (*r*), as a function of the hydration level for mainly di-18:3 MGDG, mainly di-18:2 MGDG, di-18:1 DOPE and 16:0–18:1 POPE H_II_ phases, either in the case of *dhex* or *r*, lie on one straight line, irrespectively of the degree of the acyl chains (Ref. (Bratek et al., 2019) and explanations therein). Moreover, the values of the average surface area/MGDG in the cylinders of the H_II_ phases as a function of the hydration level also lie on one curve, irrespectively of the type of acyl chains (Ref. Bratek et al., 2019), SI). Thus, these structural parameters depend predominantly on the hydration level of the phase and not on the degree of the unsaturation of the acyl chains.

## 5 Conclusion

The analyses presented in this paper revealed:

(1) In the interfacial region of the MGDG and DGDG bilayers, the galactolipid and water molecules interact via direct H-bonds and water bridges.
(2) At the bilayer/water interface MGDG interacts with water more readily than DGDG.
(3) At the bilayer/water interface the lipid-lipid interactions are more readily formed in the DGDG than the MGDG bilayer.
(4) The disproportionally higher number of DGDG-DGDG interactions relative to the number of the DGDG H-bond donor and acceptor groups can be explained by screening the DGDG β rings from the water by the α rings. This screening results in the hydration disparity of the DGDG α and β rings and the different preferences of the rings to interact via H-bonds and water bridges.
(5) The network of inter-lipid interactions at the DGDG bilayer interface is more stable and extended than that in the MGDG bilayer. Nevertheless, a DGDG aggregate under ambient conditions does not form H_II_ phase in water spontaneously; this is most likely due to the cylindrical shape of the DGDG molecule and its large head group.
(6) In the system consisting of two MGDG bilayers separated by a water layer containing 6,750 H_2_O molecules (15 H_2_O/MGDG) a MGDG stalk structure begins to form; the structure is visible as local vertical contacts of MGDG head groups from the apposing bilayer leaflets separated by water-filled tunnels (W15 system).
(7) The number of lipid-lipid and lipid-water interactions at the interface of the water-filled tunnel of the MGDG stalk structure is similar to that of the MGDG bilayer.
(8) The number of lipid-water interactions in the locally connected regions of the MGDG stalk structure is smaller than that at the interface of the MGDG bilayer.
(9) In the locally connected regions of the MGDG stalk structure both horizontal (between lipids from the same bilayer leaflet) and vertical (between lipids from apposing bilayer leaflets) inter-lipid H-bonds and water bridges are formed.
(10) The total number of lipid-lipid horizontal and vertical interactions in the locally connected regions of the MGDG stalk structure is greater than the number of horizontal lipid-lipid interactions in the MGDG bilayer.
(11) The number of lipid-water interactions in the MGDG H_II_ phase is similar to that in the locally connected regions of the stalk structure and smaller than that in the MGDG bilayer.
(12) The number of inter-lipid H-bonds (horizontal) in the MGDG H_II_ phase is greater than in the MGDG bilayer (horizontal) and similar to the total (horizontal and vertical) number of inter-lipid H-bonds in the locally connected regions of the MGDG stalk structure. The number of water bridges in the MGDG H_II_ phase is greater than in the MGDG bilayer and moderately similar to the total (horizontal and vertical) number of water bridges in the locally connected regions of the MGDG stalk structure.
(13) From 11 to 12 one can conclude that when the nonlamellar phase is formed, the lipid-water interactions are, to some extent, replaced by lipid-lipid interactions.

## Data Availability

The original contributions presented in the study are included in the article/Supplementary Material, further inquiries can be directed to the corresponding author.
