# Supplementary material for: Lipid/water interface of galactolipid bilayers in different lyotropic liquid-crystalline phases
Source: Front Mol Biosci. 2022 Aug 15;9:958537. doi: 10.3389/fmolb.2022.958537 (PMC9423040; doi:10.3389/fmolb.2022.958537)
Supplement: Supplementary file 1 [file DataSheet1.PDF]

Lipid/water interface of galactolipid bilayers in different lyotropic liquid-crystalline phases

Supporting Information

Jakub Hryc[a], Robert Szczelina[b], Michal Markiewicz[a*2], Marta Pasenkiewicz-Gierula[a*1]

[a]Faculty of Biochemistry, Biophysics and Biotechnology, Jagiellonian University, Krakow, Poland; [b]Faculty of Mathematics and Computer Science, Jagiellonian University, Krakow, Poland

Corresponding authors:
*[1]Marta Pasenkiewicz-Gierula; e-mail: marta.pasenkiewicz-gierula@uj.edu.pl
*[2]Michal Markiewicz; e-mail: m.markiewicz@uj.edu.pl

1. <u>Construction and conformational analysis of the computer model of the di-18:3-*cis* DGDG</u>

A computer model of the DGDG (Fig. S1) molecule was created from scratch using the programme PyMol (1). In addition, for comparison, the programme CHARMM-GUI Glycolipid Modeler (2) was used to build and optimise the structure of this molecule. The molecule was parameterised in the OPLS-all atom (OPLS-AA) force field for carbohydrates (3).

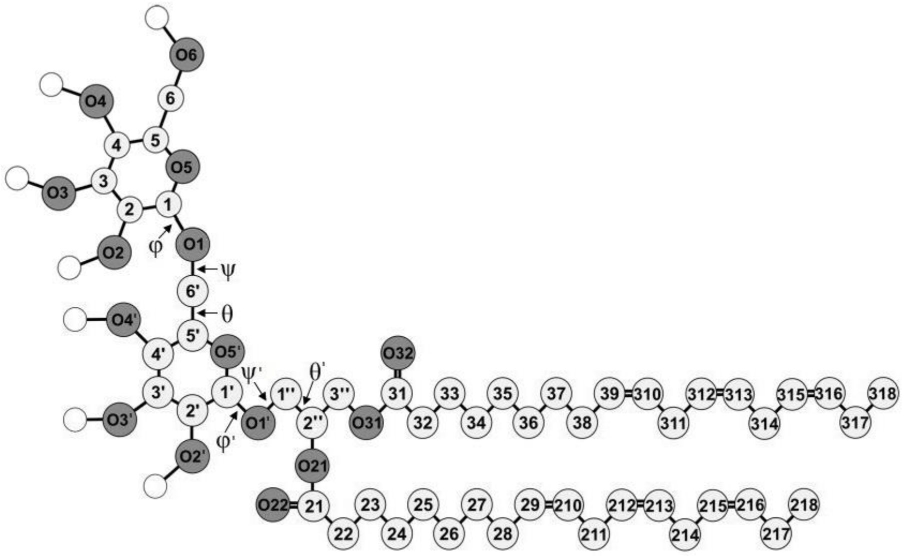

Figure S1. Molecular structure of di-18:3-*cis* DGDG. The numbering of the acyl chains and glycerol backbone atoms and notation of the torsion angle θ′ are according to Sundaralingam's nomenclature (4), with an exception for the C1″ and C3″ carbon atoms, which have been swapped here. The numbering of the galactose ring atoms and notation of the glycosidic torsion angles, φ, ψ, θ, φ′ and ψ′, are according to IUPAC convention (5). The numbers of the carbon and oxygen atoms of the β ring are marked with ′ and the C1, C2 and C3 atoms of the glycerol backbone are marked with ″ to distinguish the atoms of the α and β galactose rings from those of the glycerol backbone. Only the polar hydrogen atoms (empty circles) of DGDG are displayed; the oxygen (O) atoms are dark and the carbon atoms are light *grey*; the chemical symbol for carbon atoms, C, is omitted.

The DGDG head group consists of two galactose moieties, α-D-galactose and β-D-galactose linked by an O-glycosidic bond (linkage). The glycosidic linkage connecting the α and β rings comprises the chemical bonds connecting the C1 and O1 atoms of the α ring and the C6′ atom of the β ring (α-1-6 linkage). The glycosidic linkage connecting the β galactose and the glycerol moiety of the

galactolipid comprises the chemical bonds connecting the C1′ and O1′ atoms of the β ring and the C1″ atom of the glycerol (β-1′-1″ linkage). Conformations of both glycosidic linkages determine the orientation of the DGDG head group. The conformation of the α-1-6 linkage is defined by the values of three torsion angles, φ (O5-C1-O1-C6′), ψ (C1-O1-C6′-C5′) and θ (O1-C6′-C5′-O5′) (Fig. S1). The conformation of the β-1′-1″ linkage is defined by the values of three torsion angles φ′ (O5′-C1′-O1′-C1″), ψ′ (C1′-O1′-C1″-C2″) and θ′ (O1′-C1″-C2″-C3″) (Fig. S1).

The distributions of the values populated by the φ, ψ, and θ torsions of the α-1-6 linkage and the β-1′-1″ linkage during the last 400 ns of 1000-ns MD simulation of the DGDG bilayer at 295 K (see main text) were calculated and are shown in Fig. S2. The populated values of the angles, the percentage of a given population and the width of the population distribution are given in Table S1, together with the values for the α-1-6 linkage torsion angles of other disaccharides obtained experimentally and in other MD simulations.

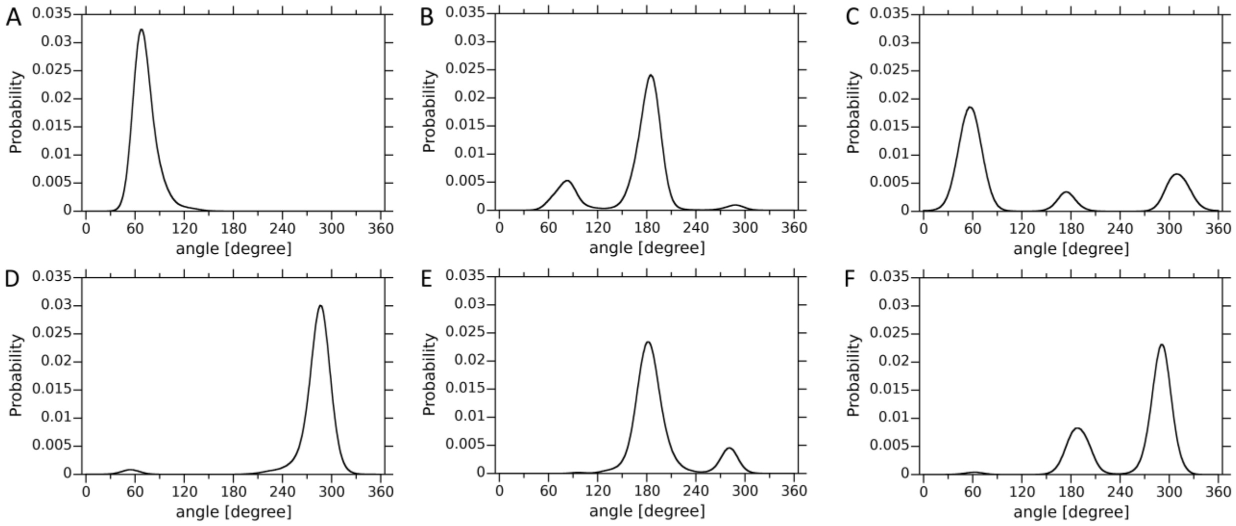

Figure S2. Distributions of (A) φ, (B) ψ and (C) θ torsion angles of the α-1-6 glycosidic linkage (between the α and β rings of DGDG) and (D) φ′, (E) ψ′ and (F) θ′ torsion angles of the β-1′-1″ glycosidic linkage (between the β ring and the glycerol backbone of DGDG) in the MD simulated DGDG bilayer at 295 K.

Table S1. Torsion angles of glycosidic bonds

**α-1-6 linkage**	φ [°] (%) (σ [°])	ψ [°] (%) (σ [°])	θ [°] (%) (σ [°])
DGDG<br>this study	68 (100) (14)	185 (79) (16)	56 (66) (17)
		84 (18) (16)	309 (24) (18)
		288 (3) (13)	174 (10) (13)
Isomaltose (5YRG, PDB)<br>(6)	69.3 ± 1.6	184.2 ± 2.8	300.2 ± 3.3
Isomaltose (MD simulation)* (7)	72;<br>78	180;<br>174	60;<br>306
Isomaltulose (IMATUL, CCDC)<br>(8)	76.9	143.6	295.4
Melibiose (MELIBM01, CCDC)<br>(9)	76.1	186.1	296.7
Melibiose (3ALT, PDB)<br>(10)	209.7 ± 5.4	172.9 ± 4.8	56.2 ± 2.4
Mannobiose (2HYQ, PDB)<br>(11)	68.8 ± 13.8	178.5 ± 24.9	63.0 ± 4.2<br>179.5± 7,8
Manα1-6Man linkage (X-ray)<br>(12)	64.7 ± 10.4;<br>67.0 ± 10.5;<br>59.4 ± 7.5	181.6 ± 10.0;<br>178.5 ± 13.7;<br>94.0 ± 17.5	59.7 ± 14;<br>186.0 ± 12.8;<br>188.5 ± 12.3
Manα1-6Man linkage (X-ray)<br>(13)	65.4 ± 9.0<br>66.5 ± 10.8<br>67.4 ± 14.4	182.6 ± 5.1<br>180.7 ± 15.1<br>109.1 ± 13.7	66.4 ± 10.2<br>185.0 ± 11.2<br>203.0 ± 22.7
**β-1′-1″ linkage**	φ′ [°] (%) (σ [°])	ψ′ [°] (%) (σ [°])	θ′ [°] (%) (σ [°])
DGDG<br>this study	286 (98) (14)	182 (87.4) (16)	291 (69) (14)
	55 (2) (13)	280 (12.2) (20)	187 (30) (17)
		97 (0.3) (12)	63 (1) (13)
MGDG<br>this study	288 (98) (15)	182 (91) (17)	291 (73) (14)
	55 (2) (17)	283 (9) (14)	188 (26) (16)
			59 (1) (15)

α-1-6 glyosidic linkage connects the α and β rings of DGDG (Fig. S1); β-1′-1″ linkage connects the β ring of DGDG or MGDG with glycerol moiety; % is the percentage of a given conformation of a torsion angle and σ is the width at half-height of a population distribution (Fig. S2). The values of the torsion angles for structures from the PDB database were obtained for disaccharides bound to proteins. *In Ref. (7), MD simulation was carried out in water.

Table S1 also includes the values of the torsion angles φ′, ψ′, and θ′ for the β-1′-1″ glycosidic linkage of DGDG and MGDG obtained in this study. The values of the β-1′-1″ linkage torsion angles and their populations for DGDG are very similar to those for MGDG.

On the basis of the entries for the α-1-6 glycosidic linkage in Table S1, one can conclude that the values of torsion angles φ, ψ, and θ, obtained in this MD simulation, concur with those published in the literature for other disaccharides.

2. Onset of new phase formation

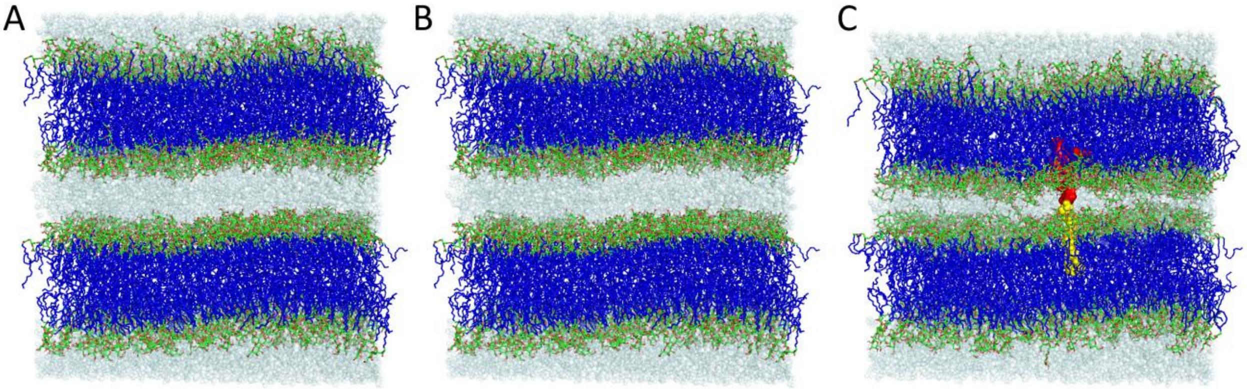

Figure S3. W15 structure (A) before and (B) after energy minimisation; (C) after the initial 1-ns of MD simulation at 295 K. The two MGDG molecules that made cross-water, vertical contact are shown in the CPK model, one is *red*, the other is *yellow*.

3. Temperature profile of W15 MD simulation

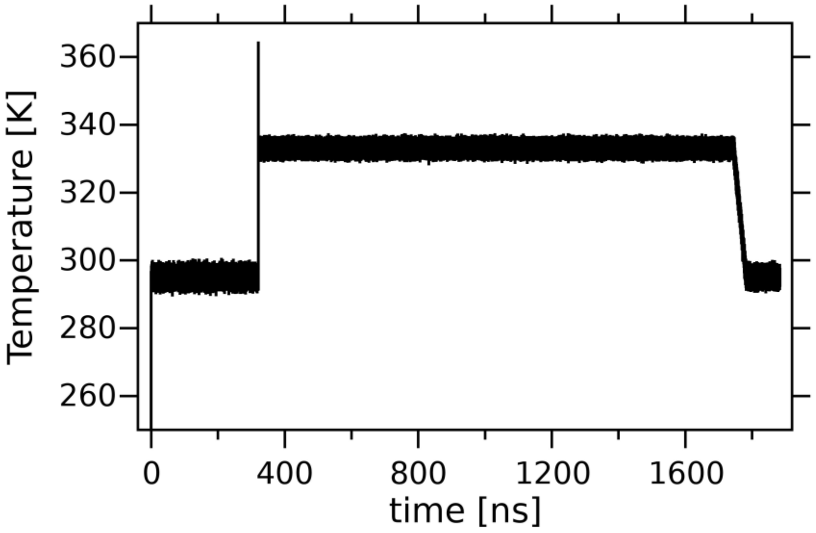

Figure S4. Time profile of the W15 temperature during the whole MD simulation of ~1.8 µs.

4. Equilibration of the DGDG bilayer

Table S2. Convergence of the DGDG bilayer parameters

DGDG	400-500 ns	500-600 ns	600-1050 ns
Ep [10^5 kJ/mol]	–2.417 ± 0.009	–2.420 ± 0.009	–2.420 ± 0.009
A_L [$Å^2$]	65.88 ± 0.60	66.67 ± 0.82	65.84 ± 0.63
R-R-distance [Å]	42.17 ± 0.33	41.86 ± 0.42	42.04 ± 0.33

Average values of the potential energy (Ep), average surface area per lipid (A_L) and bilayer width (D_{RR}) of the DGDG bilayer MD simulations at 295 K, for three time ranges, 400-500 ns, 500-600 ns, 600-1050 ns.

5. Tilt of the galactolipid rings

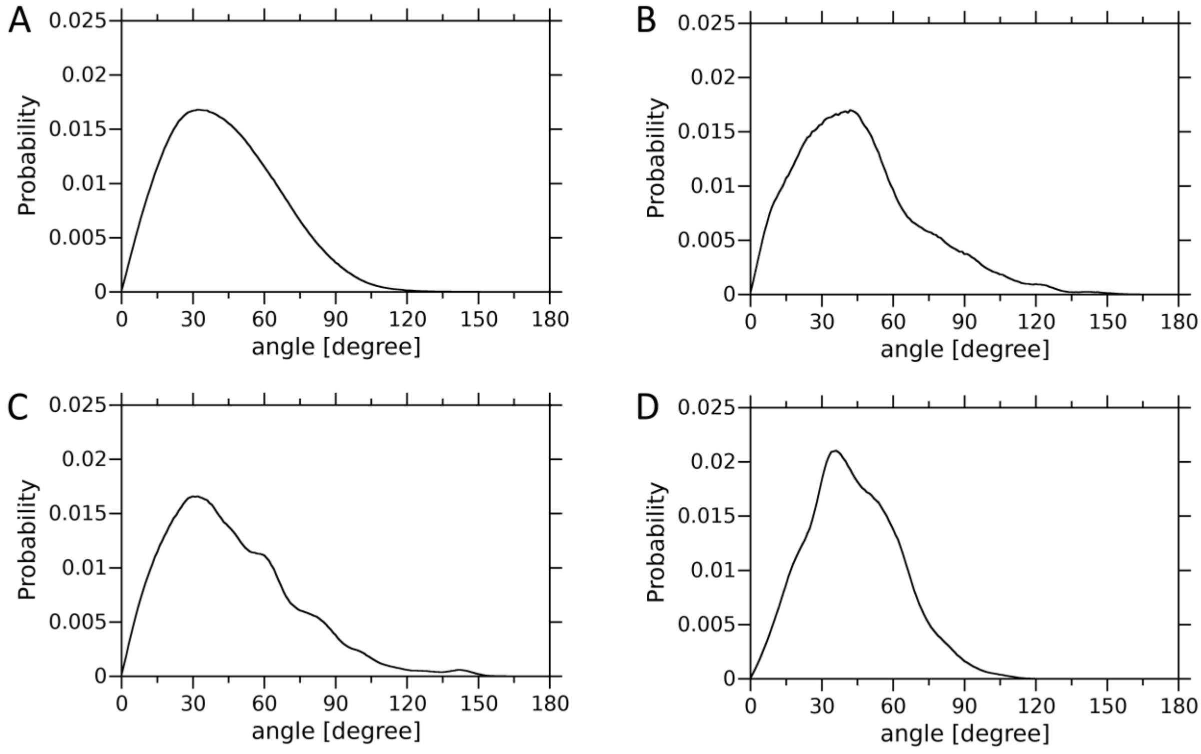

Figure S5. Distributions of ω angle (see main text) of the (A, B) MGDG β ring in the (A) MGDG bilayer and (B) flat part of W15; DGDG (C) α ring and (D) β ring in the DGDG bilayer

6. Relative orientation of the DGDG galactose rings

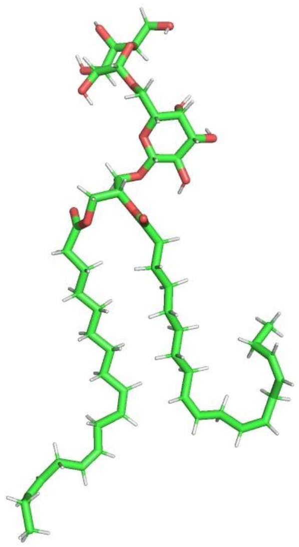

Figure S6. 3D structure of the DGDG molecule arbitrarily chosen from the bilayers showing that the α and the β ring belong to different planes.

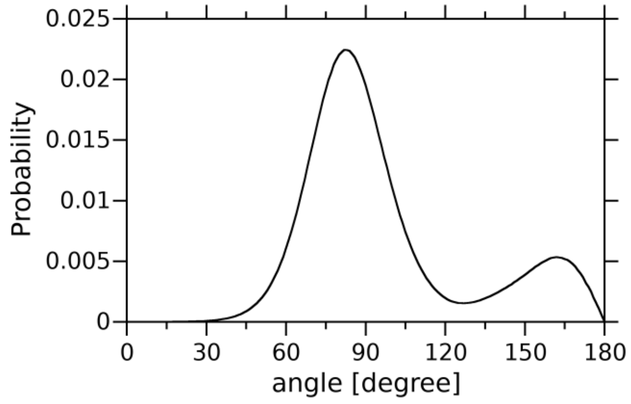

Figure S7. Distribution of angle between the α and β rings of the DGDG molecules in the DGDG bilayer. Distribution was calculated with 1° step for the last 400 ns of the 1050-ns MD simulation.

7. <u>Probability of finding terminal CH_3 of MGDG and DGDG acyl chains across the bilayer</u>

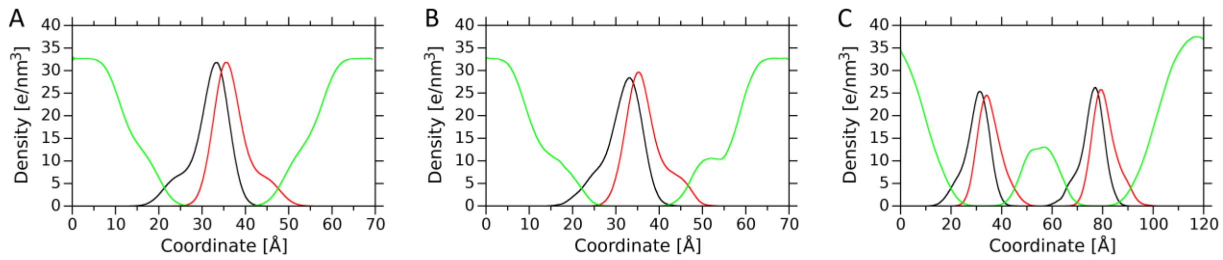

Figure S8. Electron density profiles of the terminal methyl groups (CH_3) of the galactolipid α-linolenoyl (di-18:3, *cis*) acyl chains (*red* and *black* for the "upper" and the "lower" leaflet, respectively), and the water molecules (*green*) of each leaflet across the (A) MGDG and (B) DGDG bilayers, and (C) connect-15 region of W15. The density of water was divided by 10 to show the details better.

8. Network parameters

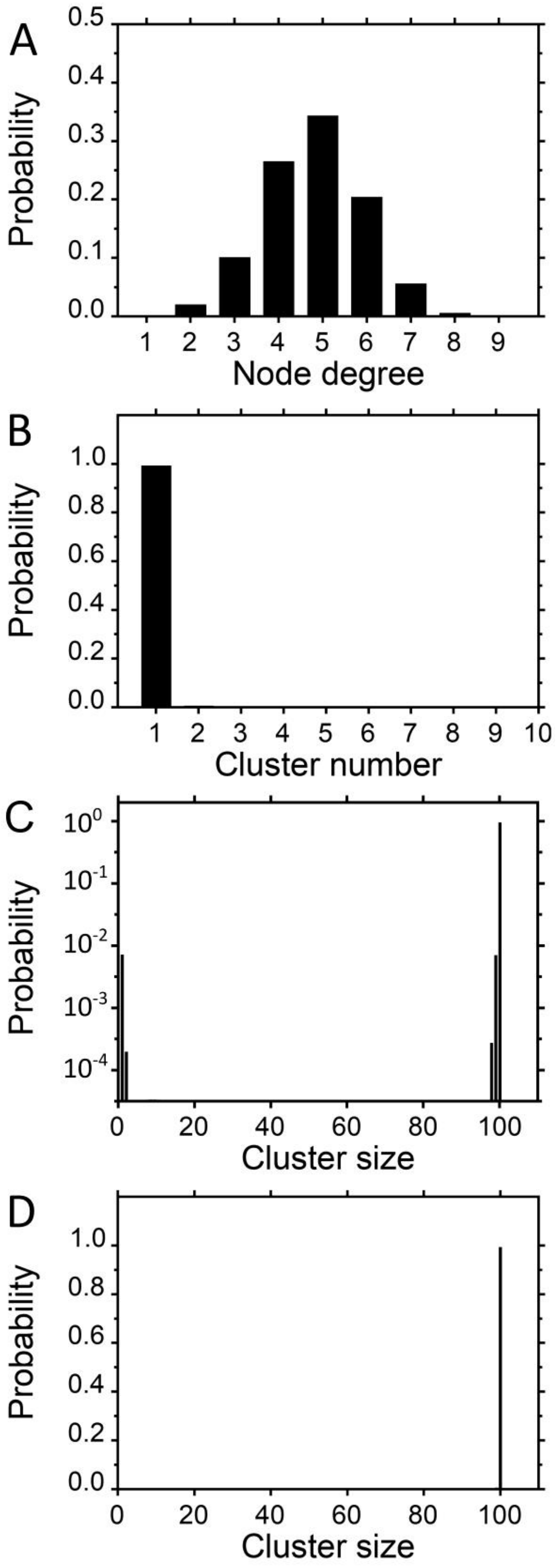

Figure S9. Distributions of (A) node degree and (B) number of clusters (cluster number); (C) logarithm of the probability of a cluster of a given size; (D) probability that an arbitrarily chosen lipid molecule belongs to the cluster of a given size, in the DGDG bilayer.

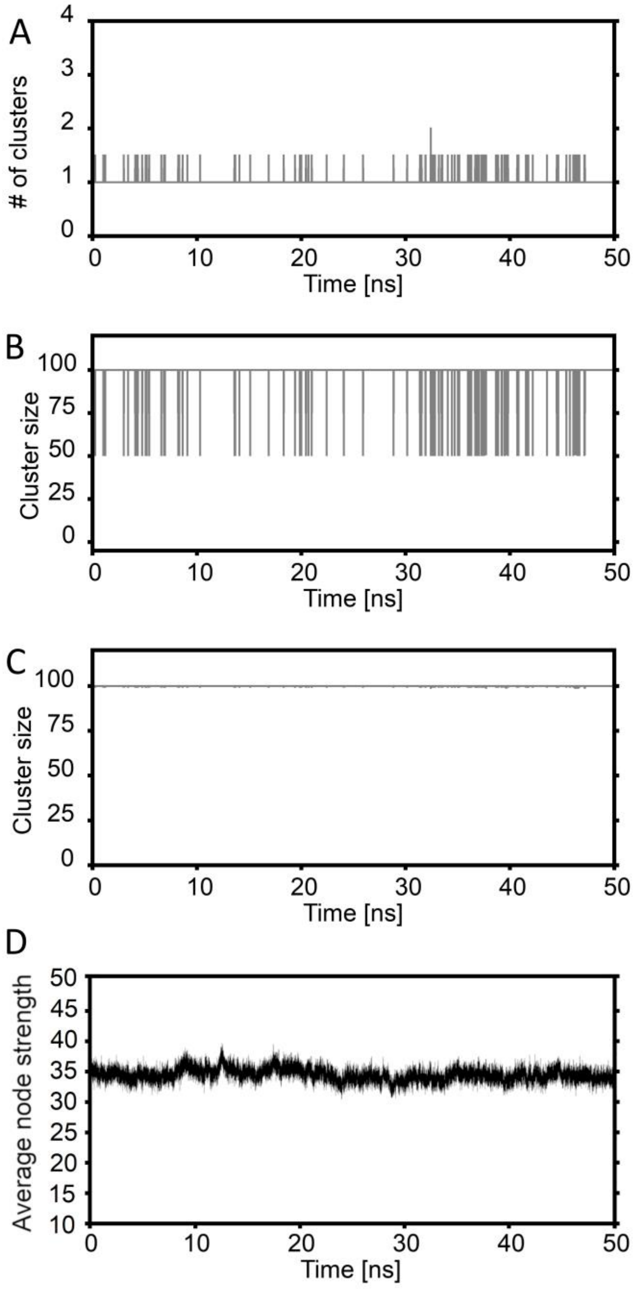

Figure S10. Time profiles of the average over two bilayer leaflets (as can be seen in Fig. S9C, there are only clusters of sizes 1, 2, 98, 99 and 100 and that of 100 is significantly more probable that the others) of the (A) number of clusters; (B) average size of the smallest cluster (the sizes of the smallest cluster are 1, 2 and 100 (Fig. S9C) – thus when in one leaflet the connected cluster breaks into two or three, the average size of the smallest cluster is ~50, otherwise it is 100); (C) average size of the largest cluster (the sizes of the largest cluster are 98, 99 and 100 – thus the average size of the largest cluster is either 100 or nearly 100); (D) average node strength, in the DGDG bilayer.

Supplementary films SF1 and SF2

Film SF1. The process of formation of the stalk structure during the initial 200-ns MD simulation (sampled with 1 ns time steps) of the double bilayer system (W15) at 295 K. The W15 system consists of two MGDG bilayers, which were initially placed parallel to each other and separated by two water layers, the thinner "inner" and the thicker "outer" layers (over the PBC). In the course of MD simulation, some regions of the apposing leaflets separated by the "inner" water layer partially dehydrate and connect locally, initiating formation of the stalk structure.

Film SF2. The dynamics of the network of lipid interconnections at the interfaces of the DGDG bilayer during 1 ns (between 950 and 951 ns) of the 1000-ns trajectory. The *black* rectangle depicts the basic simulation box. Nodes (centres-of-mass of the lipids) are presented as dots in the *x*,*y*-plane, and edges as lines connecting respective nodes. The largest cluster is in *blue* and a single-node cluster is in *orange*. To avoid problems with edges crossing PBC, 9 copies of each node are presented (strong colour tone for the cluster, soft colour tones for its 8 PBC copies); the edges are drawn in the basic simulation box and all its copies; in each time-frame (every 1 ps) the algorithm chooses the copy of the edge such that all clusters are connected components, i.e. one can trace all possible routes (paths) from a node to all other nodes in the cluster.

References

1. DeLano WL. The PyMOL Molecular Graphics System. 1,8 ed: Schrödinger, LLC; 2010.
2. Jo S, Kim T, Iyer VG, Im W. Software news and updates - CHARNIM-GUI: A web-based grraphical user interface for CHARMM. J Comput Chem. 2008;29(11):1859-65.
3. Damm W, Frontera A, TiradoRives J, Jorgensen WL. OPLS all-atom force field for carbohydrates. J Comput Chem. 1997;18(16):1955-70.
4. Sundaralingam M. Molecular structures and conformations of phospholipids and sphingomyelins. Annals of the New York Academy of Sciences. 1972;195(Jun20):324-55.
5. McNaught AD. Nomenclature of carbohydrates (IUPAC Recommendations 1996). Pure Appl Chem. 1996;68(10):1919-2008.
6. Nakae S, Shionyu M, Ogawa T, Shirai T. Structures of jacalin-related lectin PPL3 regulating pearl shell biomineralization. Proteins. 2018;86(6):644-53.
7. Peric-Hassler L, Hansen HS, Baron R, Hunenberger PH. Conformational properties of glucose-based disaccharides investigated using molecular dynamics simulations with local elevation umbrella sampling. Carbohyd Res. 2010;345(12):1781-801.
8. Dreissig W, Luger P. Die Strukturbestimmung der Isomaltulose, C12H22O11.H2O. Acta Crystallographica. 1973;B29(3):514-21.
9. Hirotsu K, Higuchi T. Conformations of Oligosaccharides .3. Crystal and molecular-structure of melibiose monohydrate. B Chem Soc Jpn. 1976;49(5):1240-5.

10. Hatakeyama T, Kamiya T, Kusunoki M, Nakamura-Tsuruta S, Hirabayashi J, Goda S, et al. Galactose recognition by a tetrameric C-type lectin, CEL-IV, containing the EPN carbohydrate recognition motif. J Biol Chem. 2011;286(12):10305-15.

11. Ziolkowska NE, Shenoy SR, O'Keefe BR, McMahon JB, Palmer KE, Dwek RA, et al. Crystallographic, thermodynamic, and molecular modeling studies of the mode of binding of oligosaccharides to the potent antiviral protein griffithsin. Proteins. 2007;67(3):661-70.

12. Wormald MR, Petrescu AJ, Pao YL, Glithero A, Elliott T, Dwek RA. Conformational studies of oligosaccharides and glycopeptides: Complementarity of NMR, X-ray crystallography, and molecular modelling. Chem Rev. 2002;102(2):371-86.

13. Petrescu AJ, Petrescu SM, Dwek RA, Wormald MR. A statistical analysis of N- and O-glycan linkage conformations from crystallographic data. Glycobiology. 1999;9(4):343-52.
